# Essential Oils for Biofilm Control: Mechanisms, Synergies, and Translational Challenges in the Era of Antimicrobial Resistance

**DOI:** 10.3390/antibiotics14050503

**Published:** 2025-05-13

**Authors:** Abdelaziz Touati, Assia Mairi, Nasir Adam Ibrahim, Takfarinas Idres

**Affiliations:** 1 Laboratoire d’Ecologie Microbienne, Université de Bejaia, FSNV, Bejaia 06000, Algeria; abdelaziz.touati@univ-bejaia.dz (A.T.); assia.mairi@univ-bejaia.dz (A.M.); 2 Department of Biology, College of Science, Imam Mohammad Ibn Saud Islamic University (IMSIU), Riyadh 13318, Saudi Arabia; 3 Laboratory for Livestock Animal Production and Health Research, Rabie Bouchama National Veterinary School of Algiers, Issad ABBAS Street, BP 161 Oued Smar, Algiers 16059, Algeria; t.idres@ensv.dz

**Keywords:** biofilms, essential oils, antimicrobial resistance, quorum sensing, extracellular matrix, synergistic therapy, nanoencapsulation, mechanisms of action

## Abstract

Biofilms, structured microbial consortia embedded in self-produced extracellular matrices, pose significant challenges across the medical, industrial, and environmental sectors due to their resistance to antimicrobial therapies and ability to evade the immune system. Their resilience is driven by multifaceted mechanisms, including matrix-mediated drug sequestration, metabolic dormancy, and quorum sensing (QS)-regulated virulence, which collectively sustain persistent infections and contribute to the amplification of antimicrobial resistance (AMR). This review critically examines the potential of plant-derived essential oils (EOs) as innovative agents for biofilm control. EOs exhibit broad-spectrum antibiofilm activity through multi-target mechanisms, including disrupting initial microbial adhesion, degrading extracellular polymeric substances (EPSs), suppressing QS pathways, and compromising membrane integrity. Their ability to act synergistically with conventional antimicrobials at sub-inhibitory concentrations enhances therapeutic efficacy while reducing the selection pressure for resistance. Despite their potential, EO applications face technical challenges, such as compositional variability due to botanical sources, formulation stability issues, and difficulties in standardization for large-scale production. Clinical translation is further complicated by biofilm stage- and strain-dependent efficacy, insufficient in vivo validation of therapeutic outcomes, and potential cytotoxicity at higher doses. These limitations underscore the need for optimized delivery systems, such as nanoencapsulation, to enhance bioavailability and mitigate adverse effects. Future strategies should include combinatorial approaches with antibiotics or EPS-degrading enzymes, advanced formulation technologies, and standardized protocols to bridge laboratory findings to clinical practice. By addressing these challenges, EOs hold transformative potential to mitigate biofilm-associated AMR, offering sustainable, multi-target alternatives for infection management and biofilm prevention in diverse contexts.

## 1. Introduction

Biofilms represent a cornerstone of microbial survival, enabling bacteria and fungi to thrive in hostile environments by forming structured, surface-adherent communities encased within a self-produced extracellular polymeric matrix [1]. These multicellular consortia are not merely passive aggregates but dynamic systems conferring significant survival advantages, including enhanced resistance to antimicrobial agents, immune evasion, and resilience against environmental stressors [2]. The ability of biofilms to colonize biotic and abiotic surfaces—ranging from human tissues to medical devices and industrial systems—underscores their clinical and environmental relevance [3]. By facilitating persistent infections, biofilm formation is intricately linked to chronic diseases, recurrent infections, and the failure of conventional therapeutic interventions, positioning biofilms as a critical challenge in modern microbiology [4].

The resistance of biofilms to treatment arises from a complex interplay of structural, physiological, and genetic factors. The extracellular matrix acts as a physical barrier, impeding drug penetration and sequestering antimicrobial agents, while the heterogeneous metabolic states of embedded cells, including dormant persister phenotypes, further reduce therapeutic efficacy. Concurrently, biofilm-associated microorganisms exhibit upregulated efflux pump activity, altered gene expression, and enhanced horizontal gene transfer, accelerating the development and dissemination of multidrug resistance (MDR). These mechanisms collectively render traditional antibiotics and antifungals insufficient, necessitating innovative strategies to disrupt biofilm integrity and restore antimicrobial susceptibility [5,6,7].

In response to these challenges, the scientific community has shifted its focus toward exploring alternative antimicrobial agents that can target biofilms through multifaceted mechanisms [8,9]. Plant-derived essential oils (EOs) have emerged as a promising frontier. These complex phytochemical mixtures exhibit broad-spectrum antimicrobial activity, disrupting biofilms through multiple pathways: destabilizing microbial membranes; inhibiting QS—a critical communication system that regulates biofilm development; and degrading extracellular matrix components [10,11]. Furthermore, EOs often exhibit synergistic effects when combined with conventional antimicrobials, thereby enhancing drug efficacy while minimizing the development of resistance [12]. Their ability to act at sub-inhibitory concentrations, natural origin, and low-toxicity profiles position them as viable candidates for standalone and adjunct therapies.

The chemical composition and antimicrobial properties of EOs are significantly influenced by geographic origin, climate conditions, and harvest-time air temperatures [13]. Studies have shown that plants’ EOs vary in chemical profile depending on whether they are grown in mountain, coastal, or inland regions [14]. This geographic variation is linked to differences in the production of key secondary metabolites, including monoterpenes, linalool, and eugenol, which contribute to aroma and bioactivity [13]. Environmental factors such as altitude, solar radiation, and water availability further affect the concentration and yield of EOs [15]. Seasonal temperature fluctuations, particularly temperature stress, have also been found to alter EO profiles [16,17]. These composition variations directly impact the oils’ antimicrobial efficacy, with certain chemotypes demonstrating enhanced activity against common pathogens like *Escherichia coli* and *Staphylococcus aureus* under specific environmental conditions [14,16].

This review article aims to analyze strategies for biofilm control using plant-derived bioactive molecules comprehensively. Given the exponential proliferation of studies in this domain over recent years, this work systematically synthesizes cutting-edge research to delineate emerging trends and evidence-based innovations. The review focuses on EOs as multifunctional antibiofilm agents. It elucidates their mechanisms of action—including quorum sensing (QS) inhibition, extracellular matrix disruption, and efflux pump modulation—while evaluating their synergies. Furthermore, it critically assesses their applicability across clinical, industrial, and environmental contexts, addressing challenges such as bioavailability and standardization. By consolidating the most recent advancements, this analysis underscores the transformative potential of natural compounds in mitigating biofilm-associated infections and antimicrobial resistance, offering a timely update to guide future research and translational applications.

## 2. Overview of Biofilms

### 2.1. Biofilm Formation

Biofilm formation is a dynamic, multistage process enabling microbial communities to adhere to biotic or abiotic surfaces, proliferate, and persist in diverse environments. The process is universally characterized by sequential phases, i.e., initial attachment, irreversible adhesion, colonization, maturation, and dispersal, with variations observed across microbial species and environmental contexts [2,18].

#### 2.1.1. Initial Attachment and Adhesion

The biofilm lifecycle begins with the reversible attachment of planktonic cells to surfaces through weak interactions, including van der Waals forces, electrostatic interactions, and hydrophobic effects. Surface properties, such as roughness, charge, and hydrophobicity, significantly influence this phase [19]. For example, hydrophobic and positively charged surfaces enhance the adhesion of *S. aureus* and oral streptococci (*Streptococcus mutans*), while negatively charged surfaces deter attachment [4,20]. Bacterial appendages, including flagella, pili, and fimbriae, mediate surface contact and overcome repulsive forces. In *Pseudomonas aeruginosa*, the Pil-Chp surface sensing system triggers the accumulation of cyclic di-GMP (c-di-GMP), transitioning cells from reversible to irreversible adhesion by upregulating adhesins and extracellular polymeric substance (EPS) production [21]. Similarly, in *Candida albicans*, adhesins such as Als1, Als3, and Hwp1 facilitate rapid attachment to both biotic (e.g., epithelial tissue) and abiotic (e.g., catheters) surfaces within 60–90 min [22].

#### 2.1.2. Colonization and Microcolony Development

Irreversible adhesion is consolidated through molecular interactions (e.g., hydrogen bonding) and EPS secretion [23]. In *S. aureus*, microbial surface components recognizing adhesive matrix molecules (MSCRAMMs), such as fibronectin-binding proteins (FnBPA/B) and clumping factors (ClfA/B), anchor cells to host tissues, while wall teichoic acids mediate attachment to abiotic surfaces [24]. EPSs, composed of polysaccharides, proteins, extracellular DNA (eDNA), and lipids, act as a structural scaffold. For instance, poly-β-(1–6)-N-acetylglucosamine (PNAG) in *Staphylococcus epidermidis* and galactosaminogalactan (GAG) in Aspergillus fumigatus stabilize microcolonies [25,26]. In *Vibrio cholerae*, cell-surface adhesion proteins (RbmA, RbmC, and Bap1) drive the transition from 2D monolayers to 3D clusters, with mechanical forces during cell division promoting vertical expansion [27,28].

#### 2.1.3. Maturation and Structural Complexity

Maturation involves the development of a stratified 3D architecture with functional and metabolic heterogeneity [29]. In oral biofilms, stratification creates oxygen and nutrient gradients, allowing anaerobic pathogens like *Porphyromonas gingivalis* to thrive in the deeper layers [20]. As observed in *P. aeruginosa* and *S. epidermidis* biofilms, water channels within the EPS matrix facilitate the distribution of nutrients and the removal of waste [25,30]. Fungal biofilms, such as those of *C. albicans*, exhibit spatial organization with yeast cells at the base and hyphae interspersed within a mannan–glucan ECM [31]. QS molecules, including acyl homoserine lactones (AHLs) and autoinducer-2 (AI-2), coordinate EPS production and metabolic specialization in multispecies communities [32,33].

#### 2.1.4. Dispersal and Recolonization

Dispersal is triggered by environmental stressors such as nutrient depletion, hypoxia, or genetic regulation [34]. In *P. aeruginosa*, the enzymatic degradation of EPSs (e.g., proteases and nucleases) and surfactants, such as rhamnolipids, facilitates the release of motile cells [35,36]. *C. albicans* dispersal involves transcriptional regulators (Nrg1 and Pes1) that promote the release of adhesive, virulent yeast cells [31]. The dispersed cells often exhibit phenotypic variations, such as antibiotic-tolerant persister cells in *S. aureus*, which enhances their survival and recolonization potential [25].

### 2.2. Regulation of Biofilms

Biofilm regulation is a multifaceted process governed by genetic, biochemical, and environmental mechanisms, enabling microbial communities to adapt to diverse conditions [37]. Central to this regulation are QS systems and second-messenger molecules, which coordinate the development of biofilms across bacterial and fungal species [38]. QS facilitates cell-density-dependent communication through signaling molecules such as acyl-AHLs in Gram-negative bacteria, autoinducing peptides (AIPs) in Gram-positive bacteria, and universal autoinducer-2 (AI-2) [39]. For instance, in *P. aeruginosa*, hierarchical QS systems (Las, Rhl, PQS, and IQS) regulate virulence factors, EPS synthesis, and dispersal enzymes. At the same time, AI-1-mediated QS in *Acinetobacter baumannii* and AI-2 in *Bifidobacterium longum* modulate biofilm formation [40,41,42]. Similarly, in *V. cholerae*, HapR-driven QS controls biofilm dispersal [43].

Cyclic di-GMP (c-di-GMP) is a pivotal second messenger that promotes biofilm stability by upregulating adhesins, pili, and EPS production while suppressing motility [44]. Elevated c-di-GMP levels in *Acidithiobacillus thiooxidans* enhance biofilm formation in acidic environments via PilZ and PelD proteins [45]. Conversely, low c-di-GMP levels in *P. aeruginosa* and *V. cholerae* favor dispersal [46]. The interplay between QS and c-di-GMP is evident in *Thermotoga maritima*, where mutual regulation optimizes biofilm dynamics [47]. Cyclic AMP (cAMP) also modulates biofilm architecture; in *P. aeruginosa*, cAMP levels influence the formation of pellicle or bottom biofilms, depending on environmental conditions [48].

In fungal biofilms, such as those formed by *C. albicans*, transcriptional networks orchestrate adhesion, matrix production, and dispersal. Six master regulators (Efg1, Tec1, Bcr1, Ndt80, Brg1, and Rob1) form an interconnected circuit that controls over 1000 target genes, including adhesins (ALS1, ALS3, and HWP1) and matrix components [31,49]. Matrix biosynthesis is further regulated by Zap1 (negative) and Rlm1 (positive), while dispersal involves Nrg1, Pes1, and chromatin-modifying complexes [50].

Environmental factors such as pH, nutrient gradients, and oxygen availability fine-tune biofilm behavior. In oral streptococci, membrane-bound sensors detect changes in redox potential and pH, triggering adaptive genetic responses [51]. *S. mutans* dominates cariogenic biofilms through aciduricity and acidogenicity [52]. Hypoxia in *P. aeruginosa* biofilms induces antibiotic tolerance by limiting the uptake of reactive oxygen species (ROS) and the proton motive force (PMF)-dependent drug [53,54]. Mechanical forces, including cellular pressure and matrix phase separation, also influence the architecture of biofilms [55]. For example, *V. cholerae* biofilms use VPS polysaccharides and proteins (RbmA, RbmC, and Bap1) to form viscoelastic hydrogels. At the same time, mechanical stress in *S. epidermidis* triggers pH-dependent matrix reorganization [7].

Persister cells further reinforce biofilm resilience, which enters dormancy via a stringent response (ppGpp) and toxin–antitoxin systems (e.g., HipBA). These cells evade antibiotics and repopulate biofilms post-treatment [56,57]. In *S. aureus*, global regulators like Agr, SarA, and SigB balance adhesin expression, protease activity, and stress adaptation. Agr-mediated dispersal via phenol-soluble modulins (PSMs) contrasts with SarA’s promotion of polysaccharide intercellular adhesin (PIA) [58].

Synthetic biology approaches have advanced biofilm regulation in engineered systems. The overexpression of cytochromes and riboflavin pathways in *Shewanella oneidensis* enhances electron transfer efficiency in bioelectrochemical biofilms [36]. Polymicrobial interactions and functional redundancy further stabilize biofilm signaling under environmental stressors, though disruptions in microbial diversity can impair QS-mediated cross-kingdom communication [32,59].

### 2.3. Functional Significance of Biofilms

Biofilms serve as critical survival structures for bacteria, enabling resilience against environmental stressors, antimicrobial agents, and host immune responses [60]. The EPS matrix, composed of polysaccharides, proteins, eDNA, and lipids, forms a multifunctional barrier that shields embedded microbial communities from physical and chemical threats [61]. For instance, biofilms reduce UV penetration, allowing only 13% of UV-C, 31% of UV-B, and 33% of UV-A to reach cells, as demonstrated in *P. aeruginosa*, *Listeria monocytogenes*, and *Deinococcus geothermalis* [18,62]. Similarly, thermophiles (*Sulfolobus acidocaldarius*) and psychrophiles (*Bacteriovorax*) rely on EPSs to buffer extreme temperatures and resist freeze–thaw cycles, while acidophiles (*Enterococcus faecalis*) and halophiles (*Halomonas stenophila*) utilize matrix components, such as inositol and 3-O-methyl glucose, to mitigate pH fluctuations and salinity stress [63,64,65,66].

A hallmark of biofilms is their ability to confer antibiotic tolerance and resistance. The EPS matrix impedes drug diffusion, binds antimicrobial agents, and facilitates enzymatic inactivation, such as that caused by β-lactamases in *Klebsiella pneumoniae* [1,67]. Additionally, reduced metabolic activity in deeper biofilm layers diminishes antibiotic efficacy, while efflux pumps and persister cells enhance survival [68,69,70]. Sub-inhibitory antibiotic concentrations further induce biofilm thickening, as observed in *S. aureus* and polymicrobial communities involving *C. albicans* and *E. coli* [71,72].

In clinical settings, biofilms drive persistent infections by evading immune recognition. EPSs mask pathogen-associated molecular patterns (PAMPs), reduce phagocytosis, and neutralize antimicrobial peptides [73]. For example, *P. aeruginosa* biofilms utilize alginate to block complement deposition, whereas *S. epidermidis* modulates immune responses through surface proteins [74,75]. Biofilms on medical devices (e.g., catheters and implants) act as reservoirs for chronic infections, necessitating device removal due to resistance to treatment [76].

Ecologically, biofilms facilitate horizontal gene transfer (HGT), promoting genetic adaptability. High cell density and matrix stability enhance conjugation, transformation, and transduction [77]. In marine systems, biofilms release metabolites that induce larval settlement in corals and macroalgae, though climate change alters these interactions [32].

Biofilms also dominate food processing environments, where pathogens such as *L. monocytogenes* and *Salmonella enterica* resist disinfectants through persister cells and EPS-mediated nutrient retention [46]. Similarly, oral biofilms (*S. mutans* and *P. gingivalis*) drive dental caries and periodontitis through acidic microenvironments and dysbiotic shifts [20].

## 3. Antibiofilm Efficacy of EOs

### 3.1. Factors Influencing Variability

Efficacy disparities in biofilm reduction by EOs arise from multiple interconnected factors, including microbial strain susceptibility, biofilm maturity, compositional oil differences, assay methodologies, and environmental conditions. Strain-specific responses are evident in examples such as Tea Tree EO (TTEO), which reduced *Xanthomonas oryzae* initial biofilm biomass to 18% at the minimum inhibitory concentration (MIC) of 18 mg/mL [78], and Coconut Oil, which exhibited starkly divergent activity: 65.48% reduction against *C. albicans* but only 15% against *S. aureus* [79]. Similarly, *P. aeruginosa* and *S. mutans* display differential resistance to the same EOs due to inherent genetic or structural adaptations [79].

Biofilm maturity critically impacts outcomes, with pre-formed biofilms often requiring higher doses for eradication than nascent biofilms. For instance, *Crithmum maritimum* EO achieved ~95% inhibition against methicillin-resistant *S. aureus* (MRSA) at ½ × MIC (MIC= 5.644 mg/mL), likely targeting metabolic pathways without bactericidal effects [80].

Methodological variability further complicates cross-study comparisons. Assay techniques such as Crystal Violet staining (measuring biomass) and log CFU reduction (viability) yield divergent results, as observed in studies of a *Cinnamon/Cardamom* EO combination tested against *E. coli* and *Bacillus subtilis* [81]. The Crystal Violet staining assay, wherein EOs are typically tested against mature biofilms formed in multi-well microtiter plates, is widely employed to quantify biofilm biomass. This method involves staining the biofilm matrix and measuring absorbance to evaluate the extent of biofilm reduction [82,83]. This assay is often used with the viable cell count method, which involves plating biofilm samples on agar plates post-treatment to count the surviving cells, providing insights into the EO’s effect on biofilm viability. This method allows researchers to assess both the reduction in biofilm mass and the viability of the cells within the biofilm [82,84].

Additionally, confocal laser scanning microscopy (CLSM) allows for the high-resolution imaging of biofilms, which in turn allows for the visualization of biofilm architecture and the penetration of EOs into biofilm layers. This method is handy for evaluating the spatial distribution and activity of EOs within the biofilm matrix, helping researchers assess how effectively the EO permeates and disrupts biofilm structure [85,86].

Furthermore, the microtiter plate assay has become a high-throughput method for assessing antibiofilm activity. In this method, biofilm formation is induced in a 96-well plate, and the EO is applied to the biofilm, with results being quantified via absorbance measurements or viable cell counts. The microtiter plate assay provides a convenient and efficient way to assess EO activity against biofilms in a controlled environment and has been widely applied in various studies [82,87].

Environmental matrices, including food models or meat substrates, alter EO activity [88]; for example, matrix effects on meat reduced EO efficacy against *Yersinia enterocolitica* [89]. The influence of the matrix on EO efficacy is typically assessed by using standard biofilm assays with or without the presence of complex matrices. For example, in food models, biofilm-forming bacteria are exposed to EOs under conditions mimicking real-world environments [89,90,91].

Nanoformulations and hybrid delivery systems enhance bioavailability and potency, exemplified by cinnamon EO–colistin nanoliposomes [92] and thymol combined with biogenic silver nanoparticles (bioAgNPs) [93], which synergistically disrupt biofilm integrity. Experimental conditions such as nanoemulsions or magnetite conjugation (e.g., *Eucalyptus globulus* EO) [94] and food-model assays (e.g., *Citrus limon* EO on kohlrabi surfaces) [91] further influence reported efficacy. These techniques enhance the penetration and effectiveness of EOs against biofilms by increasing their stability, solubility, and delivery to biofilm cells.

### 3.2. Antibiofilm Activity of EOs from Different Medicinal Plants

EOs derived from medicinal plants demonstrate significant antibiofilm activity across bacterial and fungal pathogens, with efficacy being influenced by concentration, formulation strategies, and synergistic combinations (Table 1 and Table S1 in Supplementary Data). Thyme (*Thymus* spp.), oregano (*Origanum vulgare*), and cinnamon (*Cinnamomum zeylanicum*) EOs exhibit potent activity against clinically relevant biofilms, including those formed by multidrug-resistant (MDR) strains. Innovative delivery systems, such as nanoparticle encapsulation or combinatorial therapies, further enhance their effectiveness in medical, industrial, and food safety applications.

## 4. Phytochemical Composition–Activity Correlation

EOs exhibit antibiofilm activity primarily due to dominant phytochemicals, synergism between compounds, and advanced delivery systems. Table 2 and Table S2 (Supplementary Data) summarize the key phytochemicals, their sources, inhibitory effects, and mechanisms. Phenolic monoterpenes, such as thymol and carvacrol, are among the most potent agents, disrupting microbial membranes, inhibiting QS, and degrading EPSs. For instance, *T. vulgaris* EO (75.46% thymol) achieves complete biofilm eradication in *Cutibacterium acnes* via membrane lysis and oxidative stress [103]. Synergistic combinations, such as carvacrol–p-cymene in *O. vulgare* EO, enhance penetration and gene suppression [80]. Non-phenolic terpenes, including 1,8-cineole and limonene, show moderate efficacy but require higher concentrations or formulation improvements (e.g., magnetite conjugation in *E. globulus* EO) [94]. Lower activity in oils like Sage EO (33% thujones) correlates with limited mechanistic disruption [104]. Nanoemulsions and combinatorial therapies (e.g., colistin nanoliposomes [92]) significantly amplify efficacy, underscoring the importance of delivery optimization.

## 5. Concentration-Dependent Efficacy: Thresholds and Nonlinear Responses in EO Activity

### 5.1. Thymus spp.: Sub-MIC Efficacy and Plateau Effects

*Thymus serpyllum* EO (Slovakia) demonstrated progressive biofilm inhibition, achieving a 72.93% reduction at MIC/2 (MIC_50_= 0.134 mg/mL) and more potent effects at higher doses [108]. *T. vulgaris* EO (India) reduced *C. albicans* biofilms by 80% at 0.5 × MIC (MIC= 25 µg/mL) to MIC [109], while its thymol chemotype variant (Spain) required 2 × MIC (MIC= 0.625 μL/mL) for 95.90% inhibition against unspecified strains [110]. Notably, *T. vulgaris* EO (Egypt) achieved complete biofilm eradication at 0.053 g/mL [103]. *Thymus citriodorus* (Portugal) exhibited high efficacy with Effective Concentration 50 (EC_50_) values as low as 0.062% [111], whereas *T. capitatus* EO required 0.03–0.13% *v*/*v* for total inhibition, highlighting formulation-dependent activity [100]. In contrast, *T. mastichina* (Portugal) exhibited inefficiency, requiring more than 2000% concentrations for partial biomass reduction [111]. Data gaps persist for oils like *T. vulgaris* EO (Pakistan), which reported 83.3% inhibition without detailed thresholds [112].

### 5.2. Origanum Vulgare

*O. vulgare* EO (Greece) exhibited dose-dependent biofilm reduction, increasing from 93.1% at ½ × MIC (MIC = 0.091 for MRSA strain and 0.182 mg/mL for MSSA strain) to 95.8% at 4 × MIC against *Staphylococcus* strains [80]. However, a plateau effect occurred beyond 4 mg/mL for the Kazakhstan variant [113]. Paradoxically, the Hungary-derived EO increased *Neisseria gonorrhoeae* biofilm biomass at high doses [114].

### 5.3. Cinnamomum spp.

*C. cassia* EO (China) demonstrated MIC values as low as 0.02% vol/vol against *C. auris* biofilms [99]. Cinnamon EO (Brazil) disrupted 90–100% of *S. mutans* and *S. aureus* biofilms at 4 × MIC (MIC= 0.056 mg/mL for *S. mutans* and 0.315 mg/mL for *S. aureus)* [115], while *C. verum* EO (Sri Lanka) inhibited *C. albicans* biofilms at 0.1 mg/mL [116]. Pre-formed *L. monocytogenes* biofilms required 2 × MIC (0.100 mg/mL) for eradication [117]. Sub-MIC cinnamaldehyde (Iran) reduced *E. coli* biofilm-related gene expression by 40% [118].

### 5.4. Tea Tree EOs (Maleuleka alternifolia)

TTEO achieved 100% inhibition against *S. mutans* at ≥0.5% concentration [119], while its primary component, terpinen-4-ol, showed >80% inhibition at 0.5 × MIC (MIC = 0.25% (*v*/*v*)) and near-complete eradication at 2–4 × MIC [120]. Sub-inhibitory TTEO doses reduced *X. oryzae* biofilm biomass but were less effective than MIC (18 mg/mL) [78]. Synergy with antibiotics enhanced efficacy at lower doses [121]. TTEO (Portugal) exhibited strain-specific sensitivity, with EC_50_ values ranging from 0.199% to 1.760% across the tested bacterial strains [111].

### 5.5. Eucalyptus-Derived EOs

*Eucalyptus* EO from China demonstrated dose-dependent inhibition against biofilm-forming bacteria, achieving a 77.53% reduction at 2× MIC (MIC= 15 mg/mL) [122]. Eucalyptol, a primary component of *Eucalyptus* EO, displayed potent sub-MIC activity, inhibiting biofilms at concentrations below MIC, suggesting non-bactericidal mechanisms [123]. *E. globulus* EO sustained biofilm inhibition for 24–72 h at the tested concentrations without requiring dose escalation, indicating prolonged efficacy [94]. In contrast, *Eucalyptus griffithsii* EO exhibited a linear dose–response relationship, achieving 93.73% inhibition against *S. aureus* at 20 µL/mL [124].

### 5.6. Citrus and Cymbopogon EOs

*C. limon* EO achieved 95% biofilm reduction against *N. gonorrhoeae* at 20–40 mg/mL [114], while *Citrus limetta* EO showed a linear dose–response relationship, with 0.5 mg/mL being sufficient for >90% inhibition [125]. C. *flexuosus* EO inhibited biofilms at 1 mg/mL but failed against mature biofilms at 16 mg/mL [101]. *Cymbopogon martinii* EO reduced planktonic and biofilm CFUs of *C. albicans* by 1.94 and 2.75 log10, respectively, at MIC/2 (MIC = 0.25 mg/mL) [126]. Conversely, *Cymbopogon citratus* EO fractions showed fungicidal effects only at MIC (0.16 mg/mL) levels, highlighting potential toxicity thresholds [127].

### 5.7. Clove and Other Notable EOs

Clove EO demonstrated optimal biofilm inhibition at 3% concentration, though higher doses did not enhance efficacy against unspecified bacterial strains [115]. Synergistic combinations, such as eugenol paired with mupirocin, improved biofilm reduction even at sub-MIC levels [128]. When formulated in poly-lactic acid-based nanoemulsions, carvacrol required 8% *v*/*v* for optimal activity against biofilm-forming pathogens [97].

### 5.8. Sub-MIC Efficacy and Nonlinear Responses

Eucalyptol and linalool (from *Hedychium larsenii*) inhibited *Streptococcus pyogenes* biofilms by 91% at 0.004% *v*/*v*, with effects plateauing beyond this concentration [129]. *C. verum* EO reduced *S. aureus* biofilms by 70% at 4×MIC (MIC= 0.048 mg/mL), with activity being detectable at MIC/16 [130]. However, diminishing returns were observed in citral, where 0.5% yielded ~80% biomass reduction but higher doses did not improve efficacy [131]. Similarly, lemongrass EO peaked at 0.3125% against dual-species biofilms, with no improvement at 10% [131].

### 5.9. Strain-Specific Variability

*Manuka* EO exhibited divergent effects of a 75.6% reduction in *S. aureus* biofilms at MIC/2 (MIC= 0.233 mg/mL) but no significant change against *L. monocytogenes* [132]. Sage EO showed inconsistent dose–response effects, with higher doses not constantly improving efficacy, likely due to strain-specific resistance or saturation [107]. Basil EO similarly displayed variable effects, with lower concentrations sometimes matching higher doses (e.g., 81.4–99.9% pre-adhesion inhibition) [107].

### 5.10. High-Potency and Threshold Variability

Rosemary EO from Ghana showed a clear dose–response relationship against *Salmonella* Typhi, with an IC_50_ of 2 µg/mL [133]. Garlic EO achieved 100% inhibition of *L. monocytogenes* biofilms at MIC and 2 × MIC (MIC= 0.100 mg/mL), with 68% eradication of pre-formed biofilms at MIC [117]. *Aloysia rugosa* EO required only 0.04 mg/mL for >90% inhibition [134], whereas *Cuminum cyminum* EO needed 50 mg/mL to reach 76.29% reduction [135]. *Salvia sclarea* EO achieved 87.34% biofilm reduction at 62.5 µL/L vapor concentration [136], while lavender EO required 2 × MIC for near-total biofilm removal [137].

### 5.11. Synergistic Effects of EOs with Other Substances

Combining EOs with nanoparticles, antibiotics, or advanced delivery systems has emerged as a promising strategy to overcome biofilm resistance and reduce effective doses. For instance, niosome-loaded oregano EO exhibited 2–4× greater biofilm inhibition than free EO at sub-MIC levels, attributed to enhanced bioavailability and controlled release [130]. Similarly, *O. vulgare* EO paired with biogenic silver nanoparticles (bioAgNPs) achieved 80–99% reduction in *K. pneumoniae* and *E. coli* biofilms at low sub-MIC concentrations, leveraging nanoparticle penetration and EO-mediated membrane disruption [79]. Nanoformulations, such as *Cinnamomum* EO–colistin nanoliposomes, eradicated *S. aureus* biofilms within 12 h without cytotoxicity, even at high doses [84]. Synergy with conventional antimicrobials has also been demonstrated: eugenol paired with mupirocin reduced pre-formed *L. monocytogenes* biofilms by 76.82% at sub-MIC levels, suggesting complementary mechanisms of action [85]. These combinatorial approaches enhance efficacy and mitigate risks of resistance development, positioning them as critical tools in biofilm management [79,84,130].

## 6. Toxicity and Prolonged Exposure

The clinical translation of EOs for biofilm mitigation necessitates rigorous safety assessments, as their bioactive components may exhibit dose-dependent toxicity, organ-specific risks, and route-of-administration challenges. The current literature highlights critical gaps in standardized toxicological evaluations, contrasting safety profiles among EOs, and insufficient in vivo validation, which hinder their transition to clinical or industrial applications [138,139].

### 6.1. Cytotoxicity and Organ Toxicity

Several studies report cytotoxicity associated with EO components, particularly at concentrations exceeding MICs. For instance, clove EO (eugenol-rich) demonstrated cytotoxicity to human kidney cells and zebrafish embryos at MIC × 2 concentrations (46.0 µg/mL to 102.0 µg/mL), despite efficacy against *Helicobacter pylori* [140]. Similarly, cinnamon oil nanoemulsions (CEONs) caused cytotoxicity in bovine enamel models at 5% concentration, with potential allergic reactions noted [141]. Organ-specific risks are evident in *E. globulus* EO, which induced oxidative stress and mitochondrial dysfunction in *C. albicans* but lacked safety data for human mucosal tissues [123]. Notably, thymol and carvacrol—common in *Lamiaceae* EOs—exhibited membrane disruption in vitro but raised concerns about epithelial irritation in topical applications [80,142].

### 6.2. Dose Dependency and Administration Route Risks

Dose–response relationships often reveal a narrow therapeutic window. For example, *O. vulgare* EO reduced *Salmonella* biofilm by 93–95% at ½ × MIC (0.091 mg/mL)—4 × MIC (0.182) but showed inconsistent disc diffusion results at higher doses, suggesting potential tissue damage [80]. Volatile EOs, such as rosemary and lavender EOs, demonstrated reduced efficacy in vapor phases compared with liquid formulations, with unpredictable diffusion kinetics in clinical settings [142]. Sub-inhibitory doses of caraway EO (63.7% carvone) suppressed *P. aeruginosa* virulence but failed to eradicate biofilms, risking residual pathogenicity [143]. Oral administration risks are underscored by *Syzygium aromaticum* EO (clove), which caused cytotoxicity in salivary orthodontic models at MIC levels, necessitating short-contact formulations to mitigate mucosal damage [144].

### 6.3. Lack of Standardization in Safety Protocols

Variability in EO composition due to geographic, seasonal, and extraction factors complicates safety assessments. *T. capitatus* EO exhibited chemotype-dependent toxicity, with carvacrol-dominant variants showing higher cytotoxicity than thymol-rich counterparts [145]. Similarly, *Cinnamomum verum* EO safety varied by cinnamaldehyde content (52–80%), with no consensus on acceptable thresholds for dermal or oral use [99]. Few studies quantified lethal dose (LD_50_) or no-observed-adverse-effect levels (NOAELs), and protocols for biofilm-specific toxicity (e.g., MBEC/MBIC ratios) remain non-standardized [140,146].

### 6.4. Contrasting Safety Profiles Across EO Types

Phenolic-rich EOs (e.g., thyme and oregano) consistently showed higher cytotoxicity than sesquiterpene-dominant oils. For instance, *T. vulgaris* EO caused 57–92% biofilm reduction in *S. aureus* but required ethanol emulsification to reduce epithelial toxicity [142]. In contrast, *C. limon* EO (limonene > 60%) displayed low cytotoxicity in food models, making it preferable for surface sanitation [91]. Synergistic blends, such as cinnamaldehyde–eugenol combinations, improved safety margins by lowering effective doses (FICI 0.24–0.40) compared with individual components [147]. However, *Artemisia dracunculus* EO (estragole > 60%) raised metabolite safety concerns due to potential carcinogenicity [148].

## 7. Multimodal Mechanisms of Action: Disrupting Biofilm Integrity and Microbial Physiology

**Figure 1:**
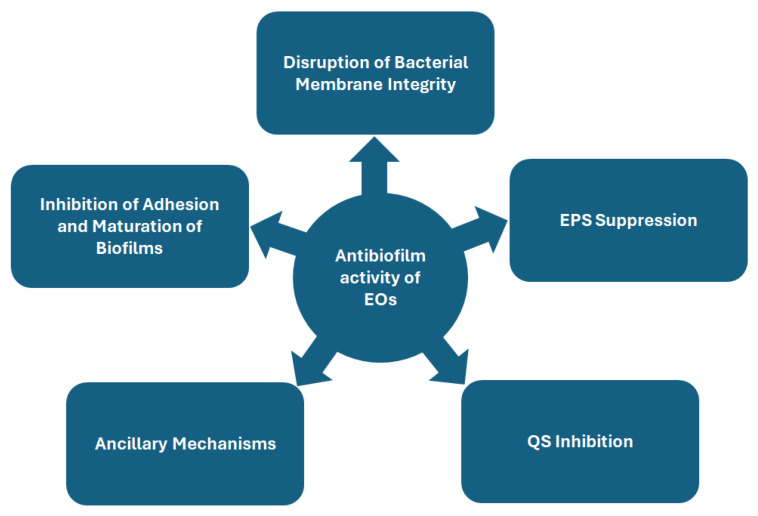
Schematic overview of proposed mechanisms of antibiofilm activity of essential oils.

Biofilms, complex microbial communities embedded in protective extracellular matrices, pose significant challenges in clinical and industrial settings due to their resilience against conventional antimicrobial therapies. Their resistance stems from dynamic adhesion processes, matrix production, QS-mediated communication, and adaptive physiological responses. Overcoming biofilm-associated infections requires innovative strategies that target multiple stages of biofilm development and microbial survival mechanisms. EOs, derived from medicinal plants, have emerged as potent antibiofilm agents due to their multifaceted, synergistic actions that disrupt structural integrity and microbial physiology (Figure 1).

### 7.1. Inhibition of Adhesion and Maturation of Biofilms

#### 7.1.1. Targeting Adhesion and Maturation: Surface Hydrophobicity, Motility, and Matrix Dynamics

EOs impede bacterial adhesion through multifaceted mechanisms, including the modulation of surface hydrophobicity, the suppression of motility-related genes, and the direct targeting of adhesion-specific proteins. *Lavandula* preparations reduced *Campylobacter jejuni* adhesion to polystyrene surfaces by ≥1 log_10_ CFU/mL at sub-inhibitory concentrations, which is linked to the downregulation of flagellar assembly genes (*motA*, *flaG*, *flgG*, *flgI*, *fliQ*, *maf4*, *pseF*, *Cj0719c*, and *Cj1467*) [137]. *Eucalyptus* spp. EOs inhibited the initial adhesion (58–95%) of *A. baumannii*, *E. coli*, *L. monocytogenes*, *P. aeruginosa*, and *S. aureus* at 10–20 µL/mL [124].

Molecular docking studies reveal that specific EO components directly interfere with adhesion proteins. For instance, eugenol (from clove EO), cinnamaldehyde (cinnamon EO), and 1,8-cineole (rosemary EO) bind strongly to the *C. albicans* Als3 adhesion protein, with eugenol exhibiting the highest binding affinity (−9.38 kcal/mol), thereby blocking fungal attachment to host surfaces [149]. In *E. coli*, heneicosane (from Jatropha intigrimma EO) disrupts FimH-mediated adhesion by interacting with residues Ile52 and Tyr48, reducing biofilm formation [150]. *Litsea cubeba* EO downregulates adhesion-related genes (*ompW*, *VP0952*, and *VP0962*) in *Vibrio parahaemolyticus*, impairing initial biofilm formation [151]. Similarly, *Ocimum basilicum* and *Salvia officinalis* EOs hinder surface colonization by reducing bacterial adhesion [107]. Thymol, a key component of thyme EO, penetrates exopolysaccharide matrices and inhibits adhesion proteins, synergizing with other compounds to block microbial attachment [152].

EOs also suppress microbial motility by targeting flagellar assembly, swarming, and QS-regulated pathways. *T. vulgaris* EO impaired motility in *P. aeruginosa*, *H. influenzae*, and *Haemophilus parainfluenzae*, inhibiting biofilm formation by 64.88–72.93% at MIC/2 (0.156 mg/mL against *H. influenzae* and *H. parainfluenzae* and 1.5–1.75 mg/mL against *P. aeruginosa)* [153]. *Plectranthus barbatus* EO (2.5% *v*/*v*) inhibits swarming and twitching motility in *P. aeruginosa* PAO1 by downregulating QS-dependent motility genes (*pil* and *fli/flh*) [154]. *L. cubeba* EO further impairs motility in *V. parahaemolyticus* by suppressing flagellar genes (*flgM*, *flgL*, *flaA*, and *flaE*) critical to movement [151]. *O. vulgare* EO reduces *P. aeruginosa* PAO1 swarming motility by 29.17% via QS interference, while *Coridothymus capitatus* EO (61% carvacrol) abolishes motility in *P. aeruginosa* at sub-inhibitory concentrations, likely through *lasR* suppression [155,156]. *O. basilicum* and *S. officinalis* EOs reduce swimming (up to 97%), twitching (up to 96.4%), and swarming motility (up to 84.9%) in bacterial strains, further hindering biofilm dispersal [107].

Additional studies highlight motility suppression in other pathogens. TTEO diminished *X. oryzae* biofilm biomass to 18 ± 1.9% by hindering swimming and swarming motility [78]. Thymus EOs reduced *S. mutans* hydrophobicity by 42.21% and inhibited biofilm formation by 98.11%, correlating with the downregulation of adhesion genes (*gbpB* and *spaP*) [110]. Laurel EO suppressed *V. parahaemolyticus* adhesion by reducing surface hydrophobicity and auto-aggregation while inhibiting swimming motility by 72% [151]. Thyme EOs reduced swimming and swarming motility in *E. faecalis* at 64–256 mg/mL, downregulating the *ebpABC* (pili formation) and *epa* (polysaccharide synthesis) genes, leading to diminished biofilm thickness [157]. *A. rugosa* EO reduced *S. mutans* adhesion to saliva-coated hydroxyapatite by 94% at 0.04 mg/mL, accompanied by decreased expression of *gbpB* and *spaP* [134].

QS-regulated swarming motility in *P. aeruginosa* PAO1 was significantly impaired by EOs. *I. verum* EO inhibited swarming by 38% at 100 µg/mL, exceeding trans-anethole activity [130]. *Anethum graveolens* EO reduced swarming by 33.33% at 100 µg/mL, outperforming limonene (28.9%) [158]. *Carum carvi* EO and its major component, carvone, exhibited dose-dependent inhibition, with 79.01% and 79.62% suppression at 2.5 mg/mL, respectively [159]. *C. cyminum* L. EO and cuminaldehyde demonstrated broad-spectrum anti-QS activity by inhibiting QS-regulated flagellar motility in *P. aeruginosa*. At 500 mg/mL, these compounds reduced motility by up to 90.12% (cumin EO) and 89.77% (cuminaldehyde), indicating their dual role in targeting both pigment production and motility [159].

#### 7.1.2. Maturation Disruption via Matrix Degradation and QS Interference

EOs disrupt biofilm maturation by degrading extracellular matrices and interfering with QS. *P. barbatus* EO inhibited *P. aeruginosa* PAO1 biofilm formation (27.87–63.6% reduction) and disrupted architecture at 2.5% *v*/*v* by suppressing swarming motility and AHL-mediated QS [154]. *Thymus spicata* EO reduced *P. aeruginosa* PAO1 biofilm biomass by 88.13% at MIC/2 (12.5 mg/mL), with carvacrol being identified as the primary component responsible for matrix disruption and QS suppression [160]. Caraway EO reduced *P. aeruginosa* PAO1 biofilm formation by 60–72% and eradicated 72–73% of mature biofilms [143].

Cinnamon-derived compounds exhibited potent maturation inhibition. *C. verum* EO eradicated 99.75% of *C. striatum* biofilms at sub-inhibitory concentrations [96], while cinnamon oil nanoemulsion (5% *v*/*v*) suppressed multispecies oral biofilm maturation, reducing red/green fluorescence ratios (0.91 ± 0.10 vs. control 1.18 ± 0.07) [141]. Clove EO reduced *Alicyclobacillus acidoterrestris* biofilm biomass by 25–65% on glass and PVC surfaces, altering spatial organization [161]. *O. vulgare* EO dispersed 70% of S. suis biofilms by disrupting cell–cell interactions [114], and *Origanum majorana* EO inhibited *C. albicans* adhesion (MBIC₉₀: 1.93 µg/mL) [162]. *C. aurantium* EO degraded *Stenotrophomonas maltophilia* and *B. subtilis* biofilms over 14 days, showing progressive structural disintegration via MALDI-TOF MS [163].

Carvacrol-loaded nanoemulsions demonstrated efficacy against mature *K. pneumoniae* biofilms by penetrating matrices and disrupting structural integrity [97]. *Nigella sativa* EO and *T. vulgaris* methanolic extract inhibited biofilm maturation in *S. aureus* (94.1% inhibition) and *P. mirabilis* (83.3% inhibition), respectively [112].

#### 7.1.3. Structural Alterations in Biofilms

EOs induce structural changes in microbial cells and biofilm architecture, disrupting their integrity and viability. Immortelle EO causes elongated cell forms in *H. influenzae*, suggesting interference with cell division [164]. Clove EO triggers cell elongation, surface roughness, and spore formation in *A. acidoterrestris* biofilms on glass and PVC surfaces [161]. Sub-inhibitory concentrations of oregano EO and carvacrol induce shrinkage and wrinkling in *E. coli* EAEC, indicative of metabolic stress [93]. TTEO and eugenol-rich *Eucalyptus* EO cause structural damage in multispecies biofilms and lead to biomass reduction and cellular scattering in *E. faecalis* and MRSA [121]. Trans-cinnamaldehyde triggers bacterial cell filamentation, forming non-viable, entangled networks [165].

Specific EOs destabilize biofilm matrices through compositional changes. Cinnamon EO modifies protein composition in *Salmonella* biofilms, weakening structural stability [163]. *Lippia origanoides* EO (thymol chemotype) disrupts *Salmonella* matrix synthesis by reducing glutamine, glutamate, and uridine diphosphate levels, impairing EPS production [166]. Linalool inhibits *S. pyogenes* biofilm formation by 91% through microcolony disruption [129]. *Centipeda minima* EO and its components (thymol and carvacrol) alter *S. aureus* cell morphology and adhesion at sub-MIC concentrations [167].

#### 7.1.4. Proteomic Alterations and Enzymatic Inhibition in Biofilms

EOs induce proteomic shifts and inhibit enzymes critical to biofilm maturation and virulence. Cedar (*Cedrus atlantica*) EO modulates protein expression in *Pseudomonas fluorescens* and *S. enterica* biofilms, altering metabolic pathways over prolonged incubation [163]. Cinnamon EO nanoemulsion (5% *v*/*v*) suppresses *C. albicans* hyphal transition by downregulating *HYR1*, *HWP1*, *ALS3*, and the *RAS1-cAMP-Efg1* pathway, reducing fungal pathogenicity [141,168]. Patchouli EO inhibits *S. mutans* glucosyltransferase activity, reducing insoluble glucan synthesis and biofilm cohesion [169].

Virulence factor inhibition is a key antibiofilm mechanism. E-cinnamaldehyde binds to *V. cholerae* biofilm-associated proteins (*RbmA*, *RbmC*, and *FabH*) and disrupts FtsZ, a cytoskeletal protein critical to cell division, leading to structural collapse [81]. *C. cyminum* L. EO and cuminaldehyde inhibit QS-regulated virulence enzymes in *P. aeruginosa*, suppressing elastase (62.12–63.14%) and protease (82.14–83.43%) production [159]. *T. citriodorus* EO reduces *C. acnes* biofilm biomass and metabolic activity, particularly in virulent phylotype IA1 strains, by altering proteomic profiles [111]. Black cardamom EO specifically targets *Salmonella* Typhimurium and *E. coli* O157:H7 biofilms (33.67–84.63% inhibition) without affecting planktonic cells, highlighting its biofilm-selective proteomic activity [170].

#### 7.1.5. Dual-Phase Activity: Adhesion and Maturation Inhibition

Several EOs exhibit dual-phase activity, targeting both adhesion and maturation stages. *Pimenta dioica* EO and its component eugenol inhibited *S. aureus* biofilms by up to 70.25% on glass surfaces [171]. Ginger EO reduced multispecies (*L. monocytogenes*, *S*. Typhimurium, and *P. aeruginosa*) biofilm adhesion at 50–100 mg/mL while altering microbial composition [172]. Garlic EO completely inhibited *S*. Typhimurium biofilm formation at MIC/2 (1/128 µL/mL) across varying temperatures [173]. Cinnamon oil suppressed *S. agalactiae* biofilm adhesion by downregulating the *pilA*, *pilB*, and *rogB* genes (0.372–0.613-fold reduction), leading to sparse biofilm structures [95]. Oregano EO (*O. vulgare*), combined with *C. ladaniferus* or *C. aurantium*, achieved 68–80% biomass reduction in *S. aureus* biofilms at sub-synergistic concentrations (1/4 MIC; MIC= 0.03% *v*/*v*) [174].

### 7.2. Disruption of Bacterial Membrane Integrity

EOs derived from medicinal plants exhibit potent antibiofilm activity by targeting bacterial membrane integrity, a critical factor in biofilm viability. This mechanism involves the physical disruption of membrane structure, increased permeability, and interference with membrane-associated functions, resulting in cytoplasmic leakage, metabolic dysfunction, and cell death.

#### 7.2.1. Membrane Structural Damage and Permeability Alterations

EOs induce structural damage and alter membrane permeability through diverse pathways. Pinus sylvestris and *C. limon* EOs caused drastic curvature changes and cytoplasmic leakage in *N. gonorrhoeae*, with 90% of treated cells appearing empty when analyzed with transmission electron microscopy (TEM). These effects were linked to lipopolysaccharide (LPS) targeting, as evidenced by the tenfold resistance of an LPS-deficient Neisseria meningitidis strain [114]. Similarly, *Helichrysum italicum* EO caused 96.1% and 79% membrane lysis in *H. parainfluenzae* and *P. aeruginosa*, respectively, at twice the MIC, accompanied by SEM-verified cell deformation [164]. Phenolic compounds, such as cinnamaldehyde in *C. zeylanicum* EO and eugenol in *E. caryophyllata* (clove) EO, directly damage bacterial cell walls, resulting in structural compromise and cellular contents leakage [115].

In *S. aureus*, *C. minima* EO and its monomers, thymol and carvacrol, elevated extracellular potassium ions (K^+^) and nucleic acids. At the same time, *T. vulgaris* EO disrupted *C. acnes* and *S. epidermidis* membranes, inducing phosphate (PO_4_^3−^) and sulfur (S^2−^) ion leakage [103,167]. Dose-dependent effects were observed in *Salmonella* Enteritidis treated with *L. origanoides* thymol chemotype EO, where 2 × MIC (MIC = 20 µL/mL) caused nucleic acid and protein leakage [166]. Similarly, *T. vulgaris* EO induced hyper-permeabilization in *Bacillus cereus*, collapsing proton pumps and depleting intracellular ATP [152]. *C. reticulata* Blanco EO caused dose-dependent membrane rupture in *L. monocytogenes*, evidenced by collapsed cell surfaces and elevated propidium iodide uptake [106].

#### 7.2.2. Hydrophobic Interactions and Lipid Bilayer Disruption

Hydrophobic EO components integrate into lipid bilayers, destabilizing membranes and triggering cytoplasmic leakage. Carvacrol in *Thymus spicata* EO disrupted *S. aureus* and *Candida parapsilosis* membranes. At the same time, D-limonene from *C. limon* EO exhibited rapid bactericidal activity against *N. gonorrhoeae* by increasing extracellular vesicle release within 15 min [114,160]. Carvacrol and thymol—major constituents of *O. vulgare* and *T. vulgaris* oils—alter membrane permeability, facilitating cytoplasmic leakage and impairing biofilm viability [115]. Thyme EO, rich in thymol, p-cymene, and γ-terpinene, induced hyper-permeabilization in *B. cereus*, collapsing proton pumps and depleting intracellular ATP [152].

*C. citratus* EO targeted ergosterol in fungal biofilms, increasing MIC values 31-fold in exogenous ergosterol, confirming membrane sterol binding as a key antifungal mechanism [127]. Similarly, *C. verum* leaf EO caused dose-dependent cell wall damage and cytoplasmic leakage in *C. albicans*, *C. tropicalis*, and *Candida dubliniensis*, corroborated by TEM showing vacuolation and scattered cytoplasmic content [116].

### 7.3. Inhibition of Biofilm Formation via EPS Suppression

EOs demonstrate potent antibiofilm activity by targeting EPS synthesis, a critical structural and functional component of biofilms. Their mechanisms include genetic regulation, enzymatic inhibition, structural disruption, biochemical depletion, and quantitative validation.

#### 7.3.1. Genetic Regulation of EPS Biosynthesis

EOs disrupt EPS production by downregulating key polysaccharide synthesis and export genes. *Lavandula* EO impedes the capsule polysaccharide biosynthesis genes *kpsM* and *kpsS* in *C. jejuni*, compromising biofilm integrity [137]. In *S. mutans*, *T. zygis* and *T. vulgaris* suppress glucosyltransferase genes (*gtfB*, *gtfC*, and *gtfD*), while *A. rugosa* EO inhibits extracellular glucan synthesis at 0.01–0.04 mg/mL [110,134]. Shuanghuanglian EO targets *wecB* and *wecC* in MDR *K. pneumoniae*, reducing O-antigen polysaccharides [175]. Anise EO reduces EPSs in *K. pneumoniae* by 92% at 90 μg/mL, disrupting fimbria and flagellum systems [176].

#### 7.3.2. Structural Disruption of Biofilm Matrices

EOs physically destabilize biofilm matrices. Linalool reduces *S. pyogenes* EPSs by 75% at 0.004% MBIC (MBIC = 0.004% (*v*/*v*)), as visualized via SEM [129]. Patchouli EO (1.25% *v*/*v*) diminishes *P. aeruginosa* PAO1 EPSs by 63.46%, disrupting *S. mutans* biofilms dose-dependently [169]. Thyme EO (128–256 mg/mL) reduces matrix density in *E. faecalis*, while oregano EO, thymol, and bioAgNP combinations collapse EAEC matrices [93,157]. *L. origanoides* EO disperses *S.* Enteritidis biofilms [166]. Thymol at sub-MIC reduces extracellular matrix density in biofilms, as observed via light microscopy, though direct biochemical validation is needed [109].

#### 7.3.3. Biochemical Depletion of EPS Components

*C. reticulata* peel EO (2% *v*/*v*) reduces *L. monocytogenes* polysaccharides, proteins, and eDNA by 54.74%, 47.77%, and 63.74%, respectively [106]. Eucalyptol decreases *Candida glabrata* carbohydrate content by 15.2–21%, alongside protein and eDNA reductions [123]. Lemongrass EO (0.3125%) and citral (0.5%) degrade biofilm matrices, reducing eDNA (86% and 63%, respectively), proteins (89% and 77%, respectively), and carbohydrates (68% and 44%, respectively) [131].

### 7.4. QS Inhibition

EOs and their bioactive constituents have demonstrated significant potential in disrupting QS-mediated biofilm formation and virulence in pathogenic bacteria. These effects are evidenced by the inhibition of QS-regulated phenotypes, such as violacein and pyocyanin production, swarming motility, and Agr system signaling, across diverse bacterial models.

Multiple EOs suppress QS-regulated violacein production in *Chromobacterium violaceum*. *O. vulgare* EO exhibited dose-dependent inhibition, achieving over 50% suppression at sub-inhibitory concentrations (MIC/4: 0.012 mg/mL) and 72.7% at MIC, outperforming its component terpinene-4-ol (42.29%) [155]. Similarly, oregano EO, carvacrol, thymol, and thymol combined with bioAgNPs reduced violacein production by 93%, 94%, 92%, and 95%, respectively, without affecting bacterial viability [93]. *I. verum* EO demonstrated 76.18% inhibition at MIC (10 mg/mL), whereas its major component, trans-anethole, showed weaker activity (48.78% at 1.25 mg/mL) [130]. *A. graveolens* EO inhibited violacein by 67.52% at MIC (10 mg/mL), outperforming its constituent limonene (18.68%) [158]. *Carum carvi* EO reduced violacein production in a concentration-dependent manner, achieving inhibition of 47.57% at MIC (10 mg/mL) and 25.28% at MIC/32 (0.312 mg/mL) [159].

Robusta coffee (*Coffea canephora*) extracts, derived from fresh and dried fruit peels, also inhibited QS in *C. violaceum*. At sub-inhibitory concentrations, these extracts reduced violacein synthesis by 44.53% to 47.48% after 24 h [177]. *C. cyminum* L. EO and its primary constituent, cuminaldehyde, suppressed violacein production in *C. violaceum* CV026 at 2 mg/mL. Cuminaldehyde exhibited stronger quantitative activity (VIC_50_ = 1.676 mg/mL) compared with the whole EO (VIC_50_ = 2.746 mg/mL) [159].

QS-regulated pyocyanin production in *P. aeruginosa* was markedly inhibited by EOs. *O. basilicum* EO reduced pyocyanin by 13.3–55.6%, while *S. officinalis* EO achieved up to 58.7% inhibition at ½ MIC (MIC ranged from 5 to 20 mg/mL) across multiple strains [107]. *C. capitatus* EO nearly abolished pyocyanin synthesis in 11 out of the 12 tested *P. aeruginosa* strains, with complete inhibition in strain 23P and 7% residual production in PA14 and 40P [156]. Although direct genetic evidence was lacking, these effects were attributed to QS interference due to the established QS control of pyocyanin [107,156].

Citral, a monoterpene aldehyde, specifically disrupted the Agr QS system in *S. aureus*. By downregulating *agrA* expression—the central regulator of the Agr system—by approximately 69.8%, citral suppressed downstream virulence pathways, including an 87.3% reduction in *hla* (alpha-toxin) expression. This inhibition highlights citral’s capacity to attenuate QS-mediated pathogenicity and biofilm stability [131].

### 7.5. Ancillary Mechanisms: Oxidative Stress, Genetic Regulation, and Host Immune Modulation

#### 7.5.1. Genetic and Metabolic Regulation

EOs modulate gene expression and metabolic pathways to impair biofilm resilience. Thymus EOs downregulate *brpA*, *vicR*, and *relA* in *S. mutans*, disrupting biofilm robustness and acid tolerance [110]. Cinnamon EO suppresses *S. agalactiae* pilus biosynthesis by downregulating *pilA*, *pilB*, and *rogB* [95], while *C. reticulata* peel EO represses *L. monocytogenes* genes involved in DNA repair (*recA* and *dnaE*), cell wall biosynthesis, and signaling (*dhaA* and *dltA*), compromising cellular integrity [106]. Artemisia EOs inhibit enteropathogenic *E. coli* (EPEC) by downregulating the LEE operon, which is critical to type III secretion systems [178]. Sub-MICs of *C. verum* and *O. majorana* EOs reduce planktonic cell viability by >90%, limiting metabolic precursors for biofilms [96]. *Lemon* EO suppresses glycolysis in *S. mutans* by reducing lactate dehydrogenase (LDH) activity and *ldh* gene expression, decreasing acid production and enamel demineralization [179]. *Artemisia* EO formulation disrupts bacterial respiration, causing dose-dependent reductions in viability (88% at 90 μg/mL) and intracellular damage [176].

#### 7.5.2. Oxidative Stress Induction and Apoptosis

EOs induce oxidative damage and apoptosis in microbial cells. Oregano EO elevates ROS and malondialdehyde levels in *E. faecalis*, causing lipid peroxidation [90]. TTEO triggers a 6-fold ROS increase in *X. oryzae* pv. *oryzae*, disrupting biofilms [78]. Eucalyptol induces ROS (a 3.1-fold increase in *C. glabrata*), disrupts mitochondrial membrane potential, and inhibits hyphal transition in *C. albicans* via *HWP1* downregulation, leading to apoptosis [123]. *Amomum villosum Lour* EO induces apoptosis-like death in MRSA, reducing viable cells from 93% to 48.8% [180]. Thymol exhibits fungicidal activity against *C. tropicalis*, reducing hyphal production and CFUs within 12–24 h [109].

#### 7.5.3. Enhanced Bioavailability via Delivery Systems

Nanoencapsulation improves EO bioavailability and antibiofilm activity. Poly(ε-caprolactone) nanocapsules (Th-NCs and Or-NCs) enable biofilm inhibition at non-cytotoxic doses [145]. Nano-gold/*L. angustifolia* composites (12.7 nm) enhance antibacterial activity and wound healing (96.78% closure), indirectly supporting biofilm control [98]. Niosome-loaded oregano EO suppresses biofilm-related genes more effectively than free oil [181]. Cinnamaldehyde-loaded nanofibers achieve rapid release (100% within 60 min) and prolonged antifungal activity against *C. glabrata* (58% CFU reduction) and *C. albicans* (49% reduction) [182].

#### 7.5.4. Anti-Inflammatory and Host-Modulatory Effects

EOs modulate host immunity and inflammation. *T. vulgaris* EO nanoemulsion reduces inflammation by 80% and NF-κB levels in a murine acne model, outperforming clindamycin in efficacy [103]. *Zanthoxylum rhoifolium* EO reduces gingival bleeding via cyclooxygenase inhibition [183]. TTEO reduces matrix metalloproteinase-8 levels in gingival crevicular fluid by 82.8%, correlating with suppressed pro-inflammatory cytokines (TNF-α, IL-1β, and IL-8) [184].

#### 7.5.5. Synergistic and Multifaceted Actions

EOs exhibit synergistic effects against multispecies biofilms. Linalool reduces *S. pyogenes* biofilm hydrophobicity (48% vs. 92% control), upregulates protease SpeB, and downregulates virulence genes (*mga* and *hasA*) [129]. Garlic EO alters multispecies biofilm composition, suppressing *L. monocytogenes* while favoring *S.* Typhimurium and *P. aeruginosa* [172]. *C. limon* EO combined with *Lactobacillus pentosus* metabolites upregulates biofilm repressor *sinR* (2.099-fold) and downregulates matrix promoters *spo0A* and *calY* (0.314–0.238-fold) in *B. cereus* [185].

## 8. Synergistic Effects of EOs in Biofilm Inhibition

### 8.1. Synergy with Enzymes

The combination of EOs with enzymes enhances biofilm disruption. For example, *Pinus sylvestris* and *C. limon* EOs co-administered with DNase I synergistically degraded *N. gonorrhoeae* biofilms, achieving >95% biomass dispersal compared with ≤60% with individual agents. This synergy stems from DNase I degrading eDNA, which otherwise impedes EO penetration. Notably, no such effect was observed when these EOs were paired with *O. vulgare* or *C. cassia* [114].

### 8.2. Synergy with Antimicrobial and Antifungal Agents

EOs enhance the efficacy of antibiotics, antifungals, and antimicrobial agents by reducing resistance and improving penetration. Cinnamon EO lowered colistin’s MIC from 16 µg/mL to 2 µg/mL against *S. aureus*, increasing inhibition zone diameters from 6.0 mm to 38.0 mm (Fractional Inhibitory Concentration Index (FICI) = 0.4) [92]. T. vulgaris EO synergized with chlorhexidine gluconate against S. mutans (FICI = 0.25) [115]. Nanoformulated C. zeylanicum EO and its cinnamaldehyde-rich fraction reduced fluconazole’s FICI to 0.26 against resistant C. auris strains, improving survival in Galleria mellonella models [105]. Citral combined with fluconazole reduced azole-resistant *C. albicans* biofilm metabolic activity (FICI = 0.5) [186]. T. vulgaris EO and thymol further synergized with fluconazole and amphotericin B, reducing MICs 32-fold against resistant Candida spp. (FICI ≤ 0.156) [109]. *M. alternifolia* and *E. globulus* EOs restored vancomycin sensitivity in Enterococcus faecium, enabling efficacy at 500-fold lower concentrations [121].

### 8.3. Advanced Delivery Systems and Mechanisms of Synergy

Nanoparticles enhance EO delivery and biofilm penetration. Oregano derivatives (thymol and carvacrol) combined with bioAgNPs reduced *E. coli* and EAEC biofilm biomass by 99% [93]. Nano-gold conjugated with *L. angustifolia* EO disrupted *P. mirabilis* biofilms at 16 µg/mL via structural collapse [98]. Composite materials like PLA/Fe₃O_4_@EG coatings reduced *E. coli* and *S. aureus* biofilm CFUs 2.5–10-fold [94]. Cellulose-based materials loaded with cinnamaldehyde delayed *S. mutant*’s growth by 35 h [187].

Surfactants improve EO bioavailability. Dioctyl sodium sulfosuccinate (DSS) combined with *Pelargonium graveolens* or *Mentha piperita* EOs reduced *Candida tropicalis* biofilm inhibition concentrations 2.5–20-fold (FICI = 0.23–0.45) [188]. Niosome-encapsulated oregano EO reduced biofilm formation 2–4-fold more effectively than free oil [181], while nanoencapsulated thyme and oregano EO inhibited *S. aureus*, *E. coli*, and *C. albicans* biofilms at 0.03–0.25 mg/mL [145]. Synergy often involves membrane disruption and enhanced penetration. Thymol with bioAgNPs suppressed *C. violaceum* QS by 95% [93]. Nanoencapsulated cinnamon oil and colistin-lysed *S. aureus* biofilms form “ghost cells” [92].

### 8.4. Synergistic Combinations and Whole-EO Efficacy

Whole EOs often outperform isolated components. *Myrtus communis* EO (containing myrtenyl acetate, 1,8-cineole, α-pinene, and linalool) inhibited *E. coli* at 6 mg/mL, outperforming individual constituents [189]. Similarly, *O. vulgare* and *Illicium verum* EOs exhibited broader activity than their isolated terpenes [130,155].

Clove and cinnamon EOs reduced MIC values in *S. aureus* (0.0156/0.0078 mg/mL) and *S. epidermidis* (0.0625/0.0312 mg/mL) via interactions between eugenol and cinnamaldehyde [190]. *O. vulgare* paired with *C. ladaniferus*, *C. aurantium var. amara*, or *Juniperus communis* showed synergy (FICI = 0.312–0.5) against *S. aureus* [174].

## 9. Application of Drug Nano-Delivery Technology in EOs

### 9.1. Nanoemulsions for Enhanced Solubility and Stability

Nanoemulsions, submicron oil-in-water emulsions stabilized by surfactants, are widely employed to overcome the hydrophobicity and volatility of EOs. By reducing the droplet size to 50–200 nm, nanoemulsions increase the surface area of EOs, enhancing their dispersion, dissolution rate, and bioavailability [191]. This nanostructuring protects EOs from oxidation, evaporation, and degradation, prolonging shelf life while improving penetration through biological membranes for topical and transdermal delivery [192]. For instance, thyme, lavender, and TTEO nanoemulsions exhibit superior antimicrobial activity due to enhanced interaction with microbial cell membranes, effectively inhibiting biofilm-forming pathogens. Similarly, *Eucalyptus* and peppermint oil nanoemulsions enable sustained anti-inflammatory effects in conditions like arthritis by facilitating deeper tissue penetration and controlled release [193,194,195]. A notable example is the clove oil nanoemulsion (eugenol ≥ 50%), which reduced *S. aureus* biofilm biomass by 52% at sub-MICs, outperforming free EO by improving eugenol’s bioavailability and prolonging antibacterial action [196].

### 9.2. Lipid-Based Nanocarriers: Liposomes, SLNs, and NLCs

Lipid-based systems, including liposomes, solid lipid nanoparticles (SLNs), and nanostructured lipid carriers (NLCs), address the instability and poor solubility of EOs [197]. Liposomes, composed of phospholipid bilayers, encapsulate both hydrophilic and hydrophobic compounds, protecting EOs like thyme and lavender from oxidative degradation while enhancing skin penetration [198]. SLNs and NLCs, with solid lipid cores, provide controlled release and long-term stability. For example, SLNs loaded with *Nigella sativa* or clove oil improve antibacterial efficacy through sustained release. NLCs co-encapsulating EOs with flavonoids (e.g., curcumin) achieve synergistic antimicrobial effects against MDR pathogens. These systems also enhance food preservation and cosmetic applications by reducing EO volatility and toxicity [194,199,200]. One study demonstrated that cinnamon oil nanoliposomes and colistin eradicated *S. aureus* biofilms through enhanced cellular uptake and synergistic interactions [92]. Similarly, lavender essential oil conjugated with gold nanoparticles eliminated *P. mirabilis* biofilms at 16 µg/mL, leveraging nanoparticle-enhanced cellular uptake while reducing cytotoxicity [98].

### 9.3. Polymeric Nanoparticles for Targeted Delivery

Biopolymeric nanoparticles, such as chitosan, poly(lactic-co-glycolic acid) (PLGA), and cellulose derivatives, offer biodegradability and biocompatibility for EO delivery [201]. Chitosan nanoparticles crosslinked with tripolyphosphate stabilize EOs like clove and peppermint, enhancing antimicrobial activity against *S. aureus* and *E. coli* via electrostatic interactions with bacterial membranes [196]. PLGA nanoparticles enable sustained release of curcumin and resveratrol, reducing oxidative stress in arthritis models [202]. Cellulose nanocrystals (CNCs) improve thyme oil stability and prolong antimicrobial action [203]. For instance, the encapsulation of lemongrass EO (citral-rich) into chitosan microparticles enhanced antifungal efficacy against *C. albicans* biofilms, achieving 84% biomass reduction at 8 × MIC due to improved mucoadhesion and controlled release [204].

### 9.4. Antimicrobial Applications Against MDR Pathogens

Nano-delivery systems enhance EO efficacy against MDR bacteria and fungi [205]. Nanoemulsions of *C. zeylanicum* and *T. vulgaris* oils disrupt fungal cell walls [99], while liposomal *Eucalyptus camaldulensis* EO combats *C. albicans* [92]. Synergistic combinations of EOs (e.g., carvacrol and thymol) with antibiotics restore sensitivity in resistant strains by inhibiting efflux pumps and biofilm formation [93]. Ethosomes and silver nanoparticles (AgNPs) conjugated with EOs such as clove and cinnamon further enhance penetration and biofilm disruption [92,95]. A study combining cinnamon essential oil with biogenic silver nanoparticles reduced MBIC_50_ values against MDR *S. agalactiae* 4–8-fold, disrupting EPSs and enhancing EO diffusion [95]. Similarly, oregano EO-loaded niosomes increased antibiofilm activity 2–4-fold against *Vibrio vulnificus* through improved stability under gastrointestinal conditions and controlled release [181].

## 10. Limitations and Challenges

EOs’ use as biofilm inhibitors has several limitations, including methodological constraints, biological variability, formulation issues, and gaps in mechanistic understanding. Recent studies have extensively reported these challenges, highlighting the complexities of translating EO-based antibiofilm strategies into clinical and industrial applications.

### 10.1. Methodological Limitations and In Vitro Model Constraints

A significant concern in EO research is the reliance on in vitro models, which often fail to replicate the complexity of in vivo environments or polymicrobial interactions. Many studies depend on monoculture biofilms formed by standard ATCC bacterial strains, neglecting the heterogeneity and dynamics observed in natural biofilms. Additionally, biofilm assessment methods, such as tetrazolium salt assays and microscopy, often lack critical parameters like MBIC and microbial biofilm eradication concentrations (MBECs), reducing the accuracy and reproducibility of results. For instance, using *C. zeylanicum* EO against mature *Staphylococcus schleiferi* biofilms revealed reduced efficacy in static in vitro models, demonstrating the difficulty in targeting established biofilms [206]. Similarly, many studies overlook long-term exposure and in vivo validation, limiting the clinical relevance of EO findings.

### 10.2. Chemical Variability and Standardization Issues

The chemical composition of EOs is inherently variable, influenced by factors such as plant chemotype, geographic origin, and extraction methods. This variability introduces significant unpredictability in EO activity. For example, *O. vulgare* EO showed strain-specific activity against *L. monocytogenes*, with efficacy fluctuating based on the levels of thymol and carvacrol. This variation complicates the standardization of EO formulations for consistent antibiofilm efficacy. Additionally, many studies fail to report the complete chemical profiles of EOs.

### 10.3. Efficacy Limitations and Cytotoxicity Concerns

EOs often require high concentrations to inhibit biofilm formation, raising concerns regarding their cytotoxicity. For instance, *S. aromaticum* EO exhibited moderate cytotoxicity to human cells at doses higher than 0.5% (*v*/*v*), limiting its therapeutic potential [207]. Some EOs, such as *M. piperita* EO, were found to disrupt beneficial microbiota, raising ecological safety concerns [208]. Furthermore, when used at higher concentrations, certain EOs, like TTEO, exhibited adverse effects such as skin irritation [209]. These cytotoxicity and safety issues necessitate careful dose optimization and rigorous toxicological assessments.

### 10.4. Strain-Specific Activity and Biofilm Stage Dependency

EO efficacy is highly strain-dependent and often varies across different biofilm stages. For example, *C. zeylanicum* EO showed limited effectiveness against some *E. coli* isolates, while *T. vulgaris* EO demonstrated strain-specific activity against *P. aeruginosa* biofilms but was less effective against mature biofilms. Some EOs, such as *O. vulgare* and *T. vulgaris*, have been reported to enhance biofilm formation at sub-inhibitory concentrations, complicating their therapeutic application. The diminished activity of EOs against mature biofilms, such as the reduced effectiveness of geranium EO against *C. albicans* after 72–96 h, highlights the challenges of targeting well-established biofilms.

### 10.5. Formulation and Delivery Challenges

Formulation issues, including the volatility and poor solubility of EOs, complicate their application in real-world settings. Encapsulation strategies, such as nanoemulsions, have been explored to enhance EO stability and solubility, yet some formulations, like those for *T. zygis* EO, showed only moderate efficiency. Moreover, matrix effects, such as the presence of organic matter in food matrices, can reduce EO efficacy. For example, *O. vulgare* EO exhibited weaker activity against *Salmonella* biofilms in chicken meat models compared with laboratory media. Additionally, the sensory properties of EOs, such as the pungent aroma of *A. sativum* EO, may limit their industrial applications, especially in food-related products.

### 10.6. Gaps in Mechanistic and Compositional Understanding

Despite promising results, the mechanistic understanding of how EOs inhibit biofilm formation remains incomplete. Studies have highlighted the disruption of extracellular matrix components and QS pathways as potential mechanisms. For instance, eugenol and carvacrol have been shown to disrupt biofilms of *S. mutans*, but their interactions with eDNA or matrix proteins remain poorly defined. Additionally, the synergistic effects of EOs with conventional antibiotics have been sparsely explored. However, some studies suggest enhanced antibiofilm activity when EOs are combined with nanoparticles or encapsulated in specific formulations. The absence of detailed compositional profiles in many studies, such as the lack of complete chemical analysis for L. angustifolia EO, further complicates understanding their antibiofilm actions.

### 10.7. Clinical and Industrial Translation Barriers

Translating EO-based antibiofilm therapies into clinical and industrial settings faces numerous barriers. The lack of in vivo validation for most EOs, such as *P. graveolens* EO against *Candida* spp., limits their clinical applicability. Additionally, challenges related to formulation instability, as seen with *Matricaria chamomilla* EO in creams, and sensory incompatibility, such as the alteration in taste in food products by *A. sativum* EO, further hinder the widespread use of EOs in medical and industrial contexts. Moreover, the inconsistent biofilm inhibitory effects of EOs and the need for high concentrations raise questions about their feasibility as standalone therapeutic agents.

## 11. Future Perspectives on the Use of EOs for Biofilm Inhibition

The escalating challenge of antimicrobial resistance has sparked renewed interest in EOs as promising natural alternatives for inhibiting biofilms. Their broad-spectrum antimicrobial activity, biocompatibility, and multi-target mechanisms position them as viable candidates across various sectors, including clinical, industrial, and environmental applications.

### 11.1. Clinical and Therapeutic Applications

EOs demonstrate significant potential in managing infections associated with MDR pathogens and biofilm-related diseases. *O. vulgare* and *C. cassia* EOs have effectively disrupted *P. aeruginosa* biofilms via QS inhibition and matrix degradation. *T. vulgaris* EO also shows promise in combating MRSA biofilms, with potential applications in wound dressings and catheter coatings to prevent device-associated infections.

In oral care, *M. piperita* and *S. aromaticum* EOs have demonstrated efficacy against cariogenic biofilms, particularly *S. mutans*. Formulations of EO-based mouthwashes and nanoparticle-based delivery systems could improve their retention and biofilm inhibition in oral environments. Furthermore, TTEO, used in topical formulations, offers low-toxicity solutions for chronic wound infections.

### 11.2. Food Safety and Agricultural Uses

EOs like thymol and carvacrol, derived from *T. vulgaris* and *Origanum* spp., have demonstrated robust activity against foodborne pathogens, including *L. monocytogenes* and *S. enterica*, through QS inhibition. These EOs offer eco-friendly alternatives for antimicrobial packaging, surface sanitization, and agricultural biocontrol. For instance, *C. verum* EO has been effective against biofilms from spoilage organisms on meat-processing equipment.

Developing EO-infused food packaging and spray sanitizers could enhance food preservation and improve food safety protocols. However, real-world testing in food systems and validation in industrial settings, especially regarding scalability and sensory impacts, are critical.

### 11.3. Formulation and Delivery Innovations

One of the significant challenges with EO applications is their stability and bioavailability. Nanoencapsulation techniques, such as liposomal or chitosan-based delivery systems, have shown promise in enhancing the stability and penetration of EOs into biofilms. For instance, nanoemulsified *O. vulgare* EO has proven effective against oral biofilms while reducing cytotoxicity. Additionally, EO-based nanoencapsulated formulations for medical devices have demonstrated improved biofilm inhibition, enhancing the potential for clinical applications.

Combining EOs with conventional antibiotics, such as ciprofloxacin and *C. cassia* EO, is a promising strategy to combat MDR pathogens. These synergistic formulations could reduce the effective antibiotic doses and mitigate the evolution of resistance.

### 11.4. Challenges and Future Research Directions

Despite their potential, several challenges remain in the clinical and industrial application of EOs for biofilm inhibition. The pharmacokinetics, in vivo toxicity, and biocompatibility of EOs need to be rigorously tested to ensure their safety in human applications. For example, Rosmarinus officinalis EO, although effective against acne-causing *C. acnes*, requires standardization to avoid skin irritation.

Future studies should focus on the mechanistic elucidation of biofilm formation through advanced techniques, such as proteomics and transcriptomics, to identify specific molecular targets and pathways. Moreover, research into synergistic combinations, formulation innovations such as thermoresponsive systems, and the scalability of EO applications will be critical to their widespread adoption.

## 12. Conclusions

Biofilms represent a formidable challenge in clinical and industrial settings, serving as a cornerstone of bacterial persistence and antimicrobial resistance. Their complex architecture, reinforced by extracellular matrices and adaptive mechanisms such as metabolic dormancy, efflux pump activation, and QS-mediated communication, renders them highly resistant to conventional therapies. These microbial communities are implicated in chronic and recurrent infections, particularly those associated with medical devices, where they evade immune responses and subvert antibiotic efficacy. The resilience of biofilms is further exacerbated by their capacity to harbor MDR pathogens, underscoring the urgent need for innovative strategies to disrupt their formation and persistence.

EOs have emerged as a promising alternative in this context due to their multifaceted antibiofilm and antimicrobial properties. These natural compounds exhibit broad-spectrum activity, targeting critical biofilm processes such as initial bacterial adhesion, matrix destabilization, and QS interference. Their ability to disrupt membrane integrity and impair virulence pathways, even at sub-inhibitory concentrations, positions them as effective agents against Gram-positive and Gram-negative pathogens. Notably, EOs demonstrate synergistic potential when combined with conventional antibiotics, enhancing therapeutic outcomes while mitigating the risk of resistance development—a significant advantage over single-target antimicrobials.

Despite their potential, the clinical translation of EOs faces notable limitations. Variability in chemical composition, influenced by botanical sources and extraction methods, compromises reproducibility and standardization. Furthermore, insufficient in vivo validation and stability, bioavailability, and potential cytotoxicity challenges hinder their widespread application. Current research gaps, including the lack of pharmacokinetic data and optimized delivery systems, must be addressed to ensure safety and efficacy in real-world settings.

Future advancements should prioritize the integration of EOs into multifunctional strategies. Innovations in formulation technologies—such as encapsulation, nanocarriers, and biofilm-resistant coatings—could enhance their stability and targeted delivery. Combining EOs with emerging approaches, including phage therapy, metabolic inhibitors, or immune-modulating agents, may further amplify their antibiofilm efficacy. Additionally, rigorous clinical trials and standardized protocols are essential to validating their therapeutic potential and establishing safety profiles.

In conclusion, EOs represent a valuable, naturally derived arsenal in the fight against biofilm-associated infections and antimicrobial resistance. Their multi-targeted mechanisms, interdisciplinary research, and formulation science advancements offer a sustainable pathway to revitalizing antimicrobial stewardship. By addressing existing limitations and fostering innovative synergies, EOs may ultimately redefine therapeutic paradigms, providing practical solutions for persistent infections in an era increasingly threatened by MDR pathogens.

## Figures and Tables

**Table 1:** Antibiofilm activity of some EOs.

Essential Oil	Target Microorganism(s)	Observed Effects	Reference
Cinnamon EO	*Streptococcus agalactiae* (14 MDR isolates)	MBIC_50_ = 4 μg/mL; >50% inhibition in all isolates	[95]
Cinnamon EO + AgNPs	*S. agalactiae*	MBIC_50_ range = 1/2–32/64 μg/mL; synergistic inhibition (FIC < 0.5)	[95]
*Thymus vulgaris* EO	106 MDR clinical strains (*E. coli*, *K. pneumoniae*, *Corynebacterium striatum*, and *S. aureus*)	Up to 99.51% biofilm reduction (*Salmonella*); dose dependency	[96]
Oregano EO + bioAgNP	Enteroaggregative *E. coli* 042 and KPC-producing *K. pneumoniae*	88–99% biofilm reduction (*E. coli*); 80–97% reduction (KPC)	[93]
Carvacrol nanoemulsion	*P. aeruginosa*	4-log (99.99%) CFU reduction in biofilm; dose-dependent eradication	[97]
Lavender EO + gold NPs	*Proteus mirabilis*, *K. pneumoniae*, MRSA, *E. coli*, and *A. baumannii*	Full biofilm eradication (*P. mirabilis* at 16 µg/mL)	[98]
*Cinnamomum cassia* EO	10 clinical *Candida auris* strains	Significant biofilm disruption at 0.02% *v*/*v*	[99]
*Thymus capitatus* EO	*S. enterica*, *L. monocytogenes*, and *Y. enterocolitica*	Total biofilm inhibition at MBIC (0.03–0.13% *v*/*v*); >6log_10_ CFU/cm^2^ reduction	[100]
*Cymbopogon flexuosus* EO	MRSA ATCC 43300	Biofilm formation inhibited at MBIC = 1 mg/mL	[101]
Clove EO	*L. monocytogenes* CECT 4032 and *Salmonella* Enteritidis CECT 4300	61.8% (*L. monocytogenes*) and 49.8% (*S.* Enteritidis) inhibition at MIC (=0.1 mg/mL)	[102]

Legend: AgNPs (silver nanoparticles), ATCC (American Type Culture Collection), CECT (Spanish Type Culture Collection), CFU (Colony-Forming Units), FIC (Fractional Inhibitory Concentration), KPC (*K. pneumoniae* carbapenemase), MBIC_50_ (Minimum Biofilm Inhibitory Concentration at 50% inhibition), MIC (minimum inhibitory concentration), MDR (multidrug resistant), NPs (nanoparticles), EO (essential oil), and *v*/*v* (Volume/Volume ratio).

**Table 2:** Key phytochemicals, EOs, and their antibiofilm activity.

Phytochemical	EO Source	Composition (%)	Target Pathogen	Biofilm Inhibition (%)/Outcome	Key Mechanisms	Reference
Thymol	*T. vulgaris* (Egypt)	75.46%	*C. acnes*	Complete eradication at 0.053 g/mL	Membrane lysis and oxidative stress	[103]
Carvacrol	*O. vulgare* (Greece)	78.72%	MRSA and MSSA	93.1–95.8% [½ × MIC (0.091 mg/mL)—4 × MIC (0.182 mg/mL)]	Synergy with p-cymene; QS inhibition	[80]
Cinnamaldehyde	*C. zeylanicum* (Belgium)	66.1%	*C. auris*	Complete disintegration at 0.06%	Cell wall disruption and synergy with fluconazole	[105]
1,8-Cineole	*E. globulus* (Romania)	Major component	*E. coli* and *S. aureus*	~66% (*E. coli*), ≥90% (*S. aureus*)	Magnetite-enhanced delivery and membrane disruption	[94]
Limonene	*Citrus reticulata* (China)	95.993%	*L. monocytogenes*	EPS reduction: proteins (−47.77%)	Membrane disruption and EPS synthesis inhibition	[106]
Synergistic blend	Carvacrol + poly(lactic acid) nanoliposomes	Core carvacrol	*P. aeruginosa*	4-log CFU reduction	Nanoemulsion-enhanced stability and delivery	[97]
Thujone	Sage EO (Serbia)	29.7% α-thujone	*P. aeruginosa*	84.1–99.6% (pre-adhesion)	Inconsistent dose–response and weak QS inhibition	[107]

## Data Availability

All data generated or analyzed during this study are included in this published article.

## Supplementary Materials

The following supporting information can be downloaded at: https://www.mdpi.com/article/10.3390/antibiotics14050503/s1, Table S1. Anti-biofilm Activities of Essential Oils and Their Combinations Against Different Microorganisms. Table S2. Chemical Composition and Biofilm Inhibition Efficacy of Essential Oils from Various Medicinal Plant Sources. References [210,211,212,213,214,215,216,217,218,219,220,221] are cited in the supplementary materials.
