# Supplementary material for: Essential Oils for Biofilm Control: Mechanisms, Synergies, and Translational Challenges in the Era of Antimicrobial Resistance
Source: Antibiotics (Basel). 2025 May 13;14(5):503. doi: 10.3390/antibiotics14050503 (PMC12108346; doi:10.3390/antibiotics14050503)
Supplement: Supplementary file 1 [file antibiotics-14-00503-s001.zip › antibiotics-3590725-supplementary.pdf]

Table S1. Anti-biofilm Activities of Essential Oils and Their Combinations Against Different Microorganisms

**EO**	**Target Microorganism(s)**	**Observed Effects**	***Study Model / Conditions***	**Reference**
Car + bioAgNP	Enteroaggregative *Escherichia coli* 042, KPC-producing *Klebsiella pneumoniae*	99% biofilm reduction (*E. coli*); 66–97% reduction (KPC-Kp)	*In vitro* ; combination therapy	[1]
Carvacrol nanoemulsion (X-NEs)	*E. coli*, *Acinetobacter baumannii*, Methicillin-resistant *Staphylococcus aureus* (MRSA), *K. pneumoniae*, *Pseudomonas aeruginosa*	4-log (99.99%) CFU reduction in *P. aeruginosa* biofilm; dose-dependent eradication	*In vitro* (co-culture with fibroblasts)	[2]
Cinnamomum cassia EO (CC-EO)	*Candida auris* (10 clinical strains)	Significant biofilm disruption at 0.02% v/v ($p < 0.001$)	*In vitro* (blood culture isolates)	[3]
Cinnamomum verum EO	*Candida albicans*, *Candida tropicalis*, *Candida dubliniensis*	$MBIC_{50}$: 0.1 mg/mL (*C. albicans*), 0.2 mg/mL (*C. tropicalis*, *C. dubliniensis*); fungicidal activity	*In vitro* (microdilution assay)	[4]
Cinnamomum verum EO	105 MDR clinical strains (*E. coli*, *K. pneumoniae*, *Corynebacterium*	Up to 99.75% biofilm inhibition (*C. striatum*); quorum sensing inhibition	*In vitro* (MDR clinical strains)	[5]

	striatum, *S. aureus*)			
Cinnamomum zeylanicum EO	*Staphylococcus saprophyticus*, *Staphylococcus pseudintermedius*	Up to 85% biofilm inhibition at 1×MIC (*S. saprophyticus*); 80% (*S. pseudintermedius*); 4×MIC required for preformed biofilm eradication	*In vitro* (canine otitis-derived MDR strains)	[6]
Cinnamomum zeylanicum EO	*C. auris* (10 clinical strains, fluconazole-resistant)	Complete biofilm disintegration at 0.06% vol/vol (MIC); partial at 0.002% with fluconazole synergy	*In vitro* and in vivo (Galleria mellonella larvae model)	[7]
Cinnamon EO	*Streptococcus agalactiae* (14 MDR isolates)	$MBIC_{50}$ = 4 µg/mL; >50% inhibition in all isolates	*In vitro* (MDR clinical isolates)	[8]
Cinnamon EO	*S. mutans*, *S. aureus*, *Lactococcus lactis*, *Enterococcus faecalis*	90–100% biofilm disruption at 4×MIC (*S. mutans*, *S. aureus*); comparable to chlorhexidine	*In vitro* (planktonic and biofilm assays)	[9]
Cinnamon EO	*Yersinia enterocolitica* strains Y4/1, Y9, Y14	Up to 78.59% biofilm reduction (Y9, 15°C TSB); dose-dependent inhibition	*In vitro* (temperature-controlled biofilm assays)	[10]
Cinnamon EO	*L. monocytogenes* CECT 4032	50% biofilm reduction at 0.5×MIC; 100% at MIC & 2×MIC (initial biofilms); 61%	*In vitro* (biofilm formation and preformed biofilm assays)	[11]

		reduction for preformed biofilms		
Cinnamon EO + AgNPs	*Streptococcus agalactiae*	$MBIC_{50}$ range = 1/2–32/64 µg/mL; synergistic for planktonic inhibition (FIC < 0.5)	*In vitro* (synergy testing)	[8]
Cinnamon EO Nanoemulsion	Multi-species oral biofilms (saliva-derived)	Reduced CFUs (4.81 ± 2.03 vs. 6.93 ± 0.31 control); absorbance reduction (0.29 ± 0.16 vs. 1.14 ± 0.17)	*In vitro* (simulated dental plaque)	[12]
Cinnamon EO	*E. coli*, *Bacillus subtilis*	*E. coli*: 100% (CV), 99% (XTT) biofilm inhibition; *B. subtilis*: 55% (CV), 36% (XTT)	*In vitro* (colorimetric assays)	[13]
Cinnamon EO	*C. albicans* (28 clinical isolates + ATCC90028)	Up to 80% biofilm reduction; strong dose-dependent inhibition	*In vitro* (clinical and reference strains)	[14]
Cinnamon EO	*C. albicans* clinical isolates	~70% biofilm biomass reduction at ¼ MIC; complete hemolysin inhibition	*In vitro* (clinical isolates)	[15]
Cinnamon EO	*S. aureus* (biofilm-forming clinical isolate)	40% biofilm reduction with mupirocin-CO cream; strong synergy with mupirocin	*In vitro* (biofilm assay)	[16]
Cinnamon/Cardamom EO Combination	*E. coli*, *B. subtilis*	*B. subtilis*: 98% (CV), 58% (XTT) biofilm	*In vitro* (synergy testing)	[13]

		inhibition; synergistic effect (FICI < 0.5)		
Eucalyptus EO	Not specified (same strains as previous study)	Weakest activity; no significant biofilm reduction	*In vitro*	[17]
Eucalyptus EO	*S. aureus* (biofilm-forming)	77.53 ± 7.29% reduction at 2×MIC; dose-dependent biofilm eradication	*In vitro* ; nanoemulsion formulation	[18]
Eucalyptus globulus EO	*E. coli* (ATCC 15224), *S. aureus* (ATCC 25923)	~66% biofilm reduction (*E. coli*); ≥90% (*S. aureus*) after 72 h; enhanced by magnetite conjugation	*In vitro* ; sustained inhibition over 24–72 h	[19]
Eucalyptus EO	Vancomycin-resistant-*Enterococcus* (VRE) (strains (specifics not provided)	Up to 58.76% (mono-species), 65.38% (multi-species) biofilm inhibition; dose-dependent	*In vitro*	[20]
Eucalyptus EO	*S. aureus* (biofilm-forming clinical isolate)	Weak effect when combined with mupirocin; limited dose-response	*In vitro* (combined with mupirocin)	[16]
Lavandula angustifolia nanoemulsion (+gold NPs)	*Proteus mirabilis*, *K. pneumoniae*, MRSA, *E. coli*, *A. baumannii*	Full biofilm eradication (*P. mirabilis* at 16 µg/mL); enhanced by gold NPs	*In vitro* ; nano-gold/EO formulation	[21]
Lavandula intermedia EO	*Streptococcus agalactiae*, *C. albicans*, *Lactobacillus spp*.	Significant reduction of 24h & 48h biofilms ($p < 0.05$); better selectivity for *Lactobacillus spp*.	*In vitro* ; tested on vaginal strains	[22]

Lavender EO	*Candida spp.* (*C. albicans*, *Candida glabrata*, etc.)	~75–85% biofilm reduction at MIC	*In vitro*	[23]
Lavender EO	Not specified (same strains as previous study)	Low impact in liquid/vapor forms; no biofilm eradication	*In vitro*	[17]
Lavender EO	*Campylobacter jejuni* NCTC 11168	~100% biofilm removal at 2× MIC; ~60% at 0.5× MIC	*In vitro* ; synergy with oxygenated monoterpenes	[24]
OEO + bioAgNP	Enteroaggregative *E. coli* 042, Carbapenem-resistant *K. pneumoniae*	88–99% biofilm reduction (*E. coli*); 80–97% reduction (KPC)	*In vitro* ; synergistic combination	[1]
OEO1 (Bosnia)	*Streptococcus mutans* ATCC 700610	80.89% biofilm reduction at ½×MIC; strong dose-dependent inhibition	*In vitro*	[25]
OEO2 (France)	*S. mutans* ATCC 700610	89.71% biofilm reduction at MIC; superior to OEO1 in gene suppression	*In vitro*	[25]
Oregano EO	*S. aureus* ATCC 6538, MRSA ATCC 43300, *E. coli* ATCC 10536	80% biofilm reduction (with *C. ladaniferus*); synergistic pairs effective at low sub-MIC levels	*In vitro*	[26]
Oregano EO	*Candida spp.* (*C. albicans*, *C. glabrata*, *Candida krusei*, etc.)	~90% biofilm reduction; clear dose response at MIC	*In vitro*	[23]

Oregano EO	*Y. enterocolitica* strains Y4/1, Y9, Y14	Up to 78.89% biofilm reduction (Y9 at 15°C TSB); SEM-confirmed cell damage	*In vitro* ; tested in TSB at 15°C	[10]
Oregano EO (Or-NC)	*S. aureus*, *E. coli*, *C. albicans*	*S. aureus*: 75% reduction (0.25 mg/mL); *E. coli*: 38.81% (0.06 mg/mL); dose-dependent inhibition	*In vitro* ; encapsulated nanoformulation	[27]
Oregano EO	*E. faecalis* (ATCC 29212 + clinical isolates)	73.64% biofilm reduction at 12×MIC; viability reduction (0.7% at 12×MIC)	*In vitro* ; tested on isolates from animals and milk	[28]
Origanum majorana EO	MDR clinical strains (*E. coli*, *K. pneumoniae*, *S. aureus*, etc.)	Maximum ~17% biofilm reduction; weak dose-response	*In vitro* ; tested on 108 MDR strains	[5]
Origanum majorana EO	*Candida spp.* (*C. albicans*, *C. tropicalis*, *C. krusei*, etc.)	~83% germ-tube inhibition (OMC at 0.125 µg/mL); dose-dependent biofilm inhibition	*In vitro*	[29]
OVEO	MSSA, MRSA, *S. aureus* ATCC 29213	~93.1–95.8% biofilm reduction at ½×MIC–4×MIC; clear dose-response	*In vitro* ; tested across concentrations (½×MIC–4×MIC)	[30]
OVEO	*Neisseria gonorrhoeae*, *Streptococcus suis*	70% biofilm reduction (*S. suis*, 24h); increased biofilm biomass at high doses in *N. gonorrhoeae*	*In vitro*	[31]

OVEO	*S. mutans*, *S. aureus*, *B. subtilis*, *E. coli*, *P. aeruginosa*, *C. albicans*	98% *S. mutans* biofilm inhibition at 4–10 mg/mL ($p < 0.05$); effect plateaued after 4 mg/mL	*In vitro* (biofilm assay)	[32]
OVEO	*S. mutans*, *S. aureus*	90–100% *S. mutans* biofilm reduction; 50–70% *S. aureus* reduction in toothpaste	*In vitro* (toothpaste formulation)	[9]
OVEO	*S. aureus*, *B. subtilis*, *E. coli*, *P. aeruginosa*, *L. monocytogenes*, *Vibrio vulnificus*	54.05% *S. aureus* biofilm reduction at 4×MIC; attributed to guaiacol–ρ-vinil	*In vitro*	[33]
Rosemary EO	Not specified (same strains as previous study)	Up to 88% biofilm reduction (non-emulsified); dose-dependent	*In vitro*	[17]
Rosemary EO	*Candida spp.* (*C. albicans*, *C. glabrata*, etc.)	~75–85% biofilm reduction at MIC	*In vitro*	[23]
Rosemary EO	*S. aureus* ATCC 25923, *E. coli* ATCC 25922	82.07% reduction (*S. aureus*), 71.98% (*E. coli*); dose-dependent	*In vitro*	[34]
Rosemary EO	*E. coli*, *K. pneumoniae*, MRSA, *S. mutans*, *B. subtilis*, *P. aeruginosa*, S. Typhi	IC_{50} as low as 2–7 µg/mL; synergy with antibiotics	*In vitro* ; broad-spectrum testing	[35]

Rosemary EO	*C. albicans* clinical isolates	Moderate biofilm inhibition; strong phospholipase reduction	*In vitro*	[15]
Rosemary EO	*P. aeruginosa* (ATCC 15442 + clinical isolates)	Up to 87.56% reduction (liquid phase), 43.74% (volatile phase); dose-dependent	*In vitro*	[36]
Sage EO	*Candida spp.* (*C. albicans*, *C. glabrata*, etc.)	~60–70% biofilm reduction; weak dose-response	*In vitro*	[23]
Sage EO	*P. aeruginosa* (clinical isolates)	Pre-adhesion: 84.1–99.6%; Post-adhesion: 0.4–95.7%; Mature: 81.3–98.7% reduction	*In vitro* ; tested on strong biofilm producers	[37]
Salvia fruticosa EO	MSSA, MRSA, *S. aureus* ATCC 29213	~93.1–95.4% biofilm reduction at ½×MIC–2×MIC; weaker dose-response	*In vitro*	[30]
Salvia officinalis EO	Multidrug-resistant *Salmonella enterica*	Complete biofilm removal at 5% EO (qualitative)	*In vitro* ; tested on raw cow milk isolates	[38]
Salvia sclarea EO	*P. fluorescens*, *P. citrinum*	Up to 87.34% reduction (*P. citrinum* in vapor phase); confirmed by MALDI-TOF MS	*In vitro* ; vapor and contact phases tested	[39]
TEO	16 clinical/reference S. aureus (MSSA/MRSA) strains	57–92% biofilm reduction via TTC/live-dead staining. Dose-dependent effect.	*In vitro (clinical strains)*	[17]

TEO	*S. mutans*, *S. aureus*	90–100% biofilm reduction in toothpaste. Synergistic with CHX (FIC ≤ 0.5).	*Ex vivo (toothpaste formulation)*	[9]
TEO	*Candida spp.* (*C. albicans*, *C. glabrata*, *C. krusei*, *C. parapsilosis*, *C. tropicalis*)	~90% inhibition at MIC.	*In vitro*	[23]
TEO	*Y. enterocolitica* strains Y4/1, Y9, Y14	Up to 78.14% inhibition (Y9); 0.64 log CFU/g reduction in meat. Dose-dependent effect.	*Ex vivo (meat matrix)*	[10]
TEO	*S. aureus*, *E. coli*, *C. albicans*	*S. aureus*: 91.1% (0.25 mg/mL); E. coli: 58.27% (0.06 mg/mL). Encapsulation enhances activity.	*In vitro (nanocarrier-enhanced)*	[27]
TEO	*Salmonella* Typhimurium, *Bacillus cereus* (from minced meat)	S. Typhimurium: 30.03–89.62% inhibition; *B. cereus*: 49.11–83.85% inhibition. Dose-dependent effect (10–100 µL/mL).	*In vitro (strains isolated from minced meat)*	[40]
TEO	*Haemophilus influenzae* DSM 4690, *H. parainfluenzae* DSM 8978, *P. aeruginosa* ATCC 27853	*H. influenzae*: 72.32%; *H. parainfluenzae*: 64.88%; *P. aeruginosa*: 72.93% inhibition at MIC/2. Dose-dependent effect.	*In vitro*	[41]

TEO	S. Typhimurium (MDR isolates)	~60% inhibition at MIC/2. Moderate antibiofilm activity.	*In vitro (abattoir isolates)*	[42]
TEO	*E. faecalis* R612-Z1 (antibiotic-resistant)	Significant reduction in EPS production and motility at 128–256 µg/mL. Dose-dependent suppression.	*In vitro (foodborne isolate)*	[43]
TEO × citriodorus (TC)	*C. acnes* (1 collection + 7 clinical strains)	EC_{50} Biomass: 0.062–0.210%; Metabolism: 0.092–0.154%. Dose-dependent inhibition.	*In vitro (clinical strains)*	[44]
Thy + bioAgNP	Enteroaggregative *E. coli* 042, KPC-producing *K. pneumoniae*	98–100% inhibition (*E. coli*); 62–100% reduction (KPC-Kp)	*In vitro* ; combination therapy	[1]
Thymus capitatus EO	*S. enterica* ser. Typhimurium, *L. monocytogenes*, *Yersinia enterocolitica*	Total biofilm inhibition at MBIC (0.03–0.13% v/v); >6 $\log_{10}$ CFU/cm² reduction at MBEC within 15 min.	*In vitro*	[45]
Thymus fallax EO	*S. mutans, S. sobrinus, S. sanguinis, S. salivarius, S. aureus, E. faecalis, C. albicans*	>95% inhibition at 8 µL/mL; up to 99% for *S. sobrinus* and *S. salivarius*. Dose-dependent effect.	*In vitro*	[46]
Thymus mastichina (TM)	*C. acnes* (1 collection + 7 clinical strains)	EC_{50} Biomass: 0.356–>2.000%; Metabolism: 0.636–2.428%. Limited effect on metabolism.	*In vitro (clinical strains)*	[44]

Thymus serpyllum EO	*P. aeruginosa* CCM 1959 (antibiotic-resistant)	72.93% biofilm inhibition (Crystal Violet assay). Dose-dependent inhibition over time (days 3–9).	*In vitro*	[47]
Thymus sibthorpii EO	MSSA, MRSA, *S. aureus* ATCC 29213	93.4–95.8% biofilm reduction at ½×MIC–4×MIC. Dose-dependent effect.	*In vitro*	[30]
Thymus vulgaris EO	106 MDR clinical strains (*E. coli*, *K. pneumoniae, C. striatum, S. aureus*)	Up to 99.51% biofilm reduction for *Salmonella*. Dose-dependent effect.	*In vitro (multidrug-resistant clinical isolates)*	[5]
Thymus vulgaris EO	*C. albicans, C. tropicalis*	26.3–80% inhibition at 0.5× MIC to higher doses. Disruption of hyphae and cell integrity.	*In vitro*	[48]
Thymus vulgaris EO	*Cutibacterium acnes* ATCC 6919, *Staphylococcus epidermidis* DSM 28319	Complete inhibition at 0.026 g/mL; eradication at 0.053 g/mL. Dose-dependent inhibition in MIC, MBIC, MBEC assays.	*In vitro*	[49]
Thymus zygis EO	S. Typhimurium	Planktonic: $\Delta\log_{10}$CFU = 2.15 (2MIC); Biofilm: $\Delta\log_{10}$CFU = 2.87 (4MIC). Stronger disruption at higher concentrations.	*In vitro (root canal model)*	[50]

Citrus reticulata Blanco cv. Tankan Peel EO (CPEO)	*L. monocytogenes* ATCC19115	EPS reduction: proteins (−47.77%), polysaccharides (−54.74%), eDNA (−63.74%) at 2%	*In vitro* (biofilm assay)	[51]
Mandarin EO (MEO)	*S. aureus* ATCC 25923, *E. coli* ATCC 25922	Significant inhibition of initial adhesion and mature biofilms; highest activity at 1:1 chitosan/MEO ratio	*In vitro* (biofilm inhibition assay)	[52]
Citrus aurantium EO (CAEO)	*Stenotrophomonas maltophilia, B. subtilis, Penicillium crustosum, Penicillium citrinum, Penicillium expansum*	$MBIC_{50}$: 8.47 µL/mL (S. maltophilia), 8.56 µL/mL (*B. subtilis*); complete biofilm degradation at day 14	*In vitro* (time-dependent biofilm degradation)	[53]
Citrus limon EO	*N. gonorrhoeae* (FA1090 + 7 clinical isolates), *S. suis* (P1/7 + 5 clinical isolates)	95% biofilm reduction (*N. gonorrhoeae* at 20–40 mg/mL); 40–60% reduction (*S. suis*)	*In vitro* (clinical isolates)	[31]
Essential Orange Oil (EOO)	*Bacillus cereus* RS1	52.1% (SS), 55.6% (AF), 65.3% (AL) biofilm reduction at sub-MIC doses; synergy with CFF enhances suppression	*In vitro* (synergy testing with CFF)	[54]

Citrus limetta EO (CLEO)	*E. coli, P. aeruginosa, S. aureus, S. enterica*	90.6% (*E. coli*), 92.19% (*P. aeruginosa*), 95.6% (*S. aureus*), 93.8% (*S. enterica*) biofilm reduction at 0.5 mg/mL	*In vitro* (ROS and cytotoxicity assays)	[55]
Citrus limon EO (CLEO)	*S. enterica, S. aureus, E. coli, Vibrio parahaemolyticus, Yersinia enterocolitica, Candida spp.*	Up to 93% inhibition (*Y. enterocolitica* on kohlrabi); ~66% for *S. enterica* on carrot/kohlrabi	*In vitro* (food model assays)	[56]
Cymbopogon flexuosus EO (EO14)	MRSA ATCC 43300	Biofilm formation inhibited at MBIC = 1 mg/mL; no eradication of mature biofilm at 16 mg/Ml	*In vitro* (MRSA biofilm assay)	[57]
Lemongrass EO (CMLEO)	*C. albicans* 8 (clinical strains)	Up to 84% biomass reduction (8×MIC); synergistic effect with chitosan microparticles	*In vitro* (chitosan microparticle synergy)	[58]
Lemongrass EO (LEO)	*C. albicans, C. tropicalis, S. aureus,* dual-species biofilms	~80% biomass & ~85% viability reduction (*C. albicans*/*S. aureus* at 0.3125% LEO); ~80% biomass reduction (*C. tropicalis*/*S. aureus* at 10% LEO)	*In vitro* (dual-species biofilm assay)	[59]
Citral (from *Cymbopogon flexuosus* EO)	*C. albicans, C. tropicalis, S. aureus*, dual-species biofilms	~74–82% biomass reduction; ~87–96% viability reduction at	*In vitro* (gene expression analysis)	[59]

		0.5%; downregulation of virulence genes		
Cymbopogon citratus EO Fractions F7 & F10	*C. albicans* NR-29450, *C. albicans* 141S, *C. glabrata* 44B	~66% reduction at MIC/10 (F7: citronellol/γ-dodecalactone; F10: γ-dodecalactone/geranial); fungicidal effect at MIC	*In vitro* (fungal cell disruption assays)	[60]
Lemongrass EO (CMLEO)	*C. albicans* (clinical strains)	~80% inhibition at 2048 µg/mL; enhanced efficacy with encapsulation	*In vitro* (encapsulation study)	[61]
Cymbopogon martinii EO	*Streptococcus mitis, Streptococcus sanguinis, E. faecalis* (clinical isolates)	$\Delta log_{10}CFU = 2.75$ (MIC/2) in biofilm; comparable to triple antibiotic paste (TAP)	*In vitro* (root canal biofilm model)	[50]
Citronellol	*E. coli* CECT 434	53% metabolic activity reduction; 90% culturability reduction	*In vitro* (microcolony and membrane integrity assays)	[62]
Mentha arvensis EO	Mixed microbial strains (study unspecified)	Moderate to strong reduction depending on concentration; less effective than synergistic combinations	*In vitro* (antimicrobial assay)	[22]
Peppermint EO	*C. albicans, C. glabrata, C. krusei, C. parapsilosis, C.*	~75–85% biofilm reduction at MIC	*In vitro* (EPS disruption assay)	[23]

	tropicalis, C. guilliermondii			
Agastache rugosa EO (AREO)	*S. mutans* (ATCC 25175)	>90% biofilm inhibition at 0.04 mg/mL; adhesion reduction (46.2% to 6.2% at 0.02–0.04 mg/mL)	*In vitro* (biofilm assay)	[63]
Onion EO (EOO)	*L. monocytogenes* CECT 4032	77% biofilm reduction at 0.5×MIC; 100% at 2×MIC	*In vitro* (dose-dependent assay)	[11]
Garlic EO (EOG)	*L. monocytogenes* CECT 4032	50% inhibition at 0.5×MIC (initial); 100% at MIC; 68% for preformed biofilms	*In vitro* (preformed biofilm assay)	[11]
Garlic EO (GEO)	S. Typhimurium (MDR isolates)	~80% biofilm reduction at MIC/2	*In vitro* (temperature-controlled assay)	[42]
Ammi visnaga EO	*A. baumannii, E. coli, L. monocytogenes, P. aeruginosa, S. aureus*	Up to 53.56% (*E. coli*), 33.80% (*L. monocytogenes*), 41.45% (*P. aeruginosa*); minimal effect on *S. aureus*	*In vitro* (docking and biofilm assays)	[64]
Amomum villosum Lour EO	*S. aureus* ATCC 43300 (MRSA)	Significant biofilm reduction at 1 MIC and 1/2 MIC; dose-dependent	*In vitro* (MRSA biofilm assay)	[65]
Black cardamom EO (BCEO)	*E. coli* O157:H7, S. Typhimurium, *C. albicans, S.*	*E. coli*: 47.31–84.63%; S. Typhimurium: 33.67–	*In vitro* (multi-strain biofilm assay)	[66]

	mutans, S. aureus, etc.	50.17% (dose-dependent; 0.03–0.5%)		
Anethum graveolens EO	*L. monocytogenes, Vibrio vulnificus, Shigella flexneri, B. subtilis*, etc.	>50% reduction at 1×MIC; up to 67.5% violacein inhibition (QS activity)	*In vitro* (QS inhibition assay)	[67]
Artemisia dracunculus EO	*S. aureus* ATCC 25923, S. Typhimurium ATCC 14028	Significant biofilm reduction at MIC/2 and MIC/4 ($P < 0.001$ for S. Typhimurium)	*In vitro* (dose-response assay)	[68]
Backhousia citriodora EO (BCEO)	*S. aureus, S. epidermidis, E. coli, K. pneumoniae*	Inhibition: 85.10%–96.44%; eradication: 70.92%–90.73%	*In vitro* (multi-species biofilm assay)	[69]
Caraway EO (CEO)	*P. aeruginosa* PAO1	60–72% biofilm inhibition; 72–73% eradication at 1% CEO	*In vitro* (virulence attenuation assay)	[70]
Cedar atlantica EO (CEO)	*P. fluorescens, S. enterica, C. albicans*	87% reduction for *C. albicans* at 0.01%; 100% at 0.1%	*In vitro* (protein disruption assay)	[71]
Cistus ladanifer (CL)	*C. acnes* (clinical strains)	EC_{50} Biomass: 0.083–>0.500%; Metabolism: 0.250–>0.500%	*In vitro* (clinical strain assay)	[44]
Coconut Oil	*S. aureus, E. faecalis, Streptococcus spp., C. albicans*	65.48% (*C. albicans*), 28.46% (*Streptococcus spp.*), 15% (*S. aureus*)	*In vitro* (oil-pulling model)	[72]
Crithmum maritimum EO	Methicillin-sensitive *Staphylococcus*	~91.5–95.6% biofilm inhibition at ½×MIC–2×MIC	*In vitro* (low-dose biofilm assay)	[30]

	aureus (MSSA), MRSA, *S. aureus* ATCC 29213			
Cuminum cyminum EO	*Vibrio spp.* (15 strains)	Up to 76.29% biofilm inhibition at 50 mg/mL	*In vitro* (QS and virulence enzyme assay)	[73]
Green Cardamom EO	*E. coli* O157:H7, S. Typhimurium	*E. coli*: 64.29–85.59%; *Salmonella*: 6.13–100% (dose-dependent)	*In vitro* (virulence factor assay)	[74]
Illicium verum	*S. aureus, L. monocytogenes, V. vulnificus, S. enterica*, etc.	>50% reduction at MIC; up to 70% eradication at 4×MIC	*In vitro* (surface-specific biofilm assay)	[75]
Clove EO	*L. monocytogenes* CECT 4032, S. Enteritidis CECT 4300	61.8% (*L. monocytogenes*) and 49.8% (S. Enteritidis) inhibition at MIC; up to 77.2% and 60% at 2× MIC	*In vitro* (adhesion and eradication assay)	[76]
Ginger EO	*L. monocytogenes, S. Typhimurium, P. aeruginosa*	Significant sessile reduction in 1–48 h; *L. monocytogenes* most sensitive	*In vitro* (mixed-culture biofilm assay)	[77]

Table S2. Chemical Composition and Biofilm Inhibition Efficacy of Essential Oils from Various Medicinal Plant Sources

EO	Main Compound(s) (%)	Origin	Target Pathogen	Biofilm Inhibition (%) or Outcome	Synergistic Effects/Additives	Notable Remarks	Reference
Rosemary Oil (R-EO)	1,8-Cineole (30.12%)	Poland	*Pseudomonas aeruginosa*	87.56% (liquid), 43.74% (volatile)	Synergy with other terpenes	High 1,8-cineole content drives activity	[36]
Laurel EO (LEO)	1,8-Cineole (30.8%)	Not specified	*Vibrio parahaemolyticus*	51.40% at 0.05 mg/mL	Synergistic with other terpenes	Reduces motility and gene expression	[78]
LEO (Lavender Essential Oil)	1,8-Cineole (36.1%), Linalool (15.8%)	Slovenia	*Campylobacter jejuni*	~100% at 2× MIC; ~60% at 0.5× MIC	Synergy with oxygenated monoterpenes	Anti-motility and anti-adhesion effects	[24]
Salvia officinalis EO	1,8-Cineole (39.18%)	Saudi Arabia	Multidrug-resistant *Salmonella enterica*	Complete biofilm removal (5% EO)	–	Linked to 1,8-cineole and β-caryophyllene	[38]
Thymus mastichina (TM)	1,8-Cineole (43.5%)	Portugal	*Cutibacterium acnes* (clinical strains)	EC_{50} Biomass: 0.356–>2.000%; limited metabolic inhibition	None	Less effective than geraniol; requires higher doses	[44]
Eucalyptus Oil (EEO)	1,8-Cineole (58.07%)	Spain	Vancomycin-resistant *Enterococcus* (VRE)	58.76% (mono-species), 65.38% (multi-species)	–	Best inhibition in VRE strains; dose-dependent	[20]
Eucalyptus globulus EO (EG EO)	1,8-Cineole (major); total EO loading: 0.36 ± 0.1 wt.%	Romania	*Escherichia coli*, *Staphylococcus aureus*	~66% (*E. coli*), ≥90% (*S. aureus*)	Magnetite conjugation	Enhanced stability and surface delivery; high activity attributed to 1,8-cineole	[19]
E-EO (Eucalyptus)	1,8-Cineole, γ-Terpinene	Not specified	*S. aureus*	Weakest in all assays	–	Indifferent activity when combined with mupirocin	[17]
COE/Colistin Nanoliposomes	2-propenal-3-phenyl (52.1%)	Egypt	*S. aureus*	Complete eradication with COE/colistin after 12 h	Synergy with colistin	Nanoformulations enhance dose-dependent efficacy	[79]
Eucalyptol (EPTL)	99% pure 1,8-Cineole	India	*Candida albicans*, *Candida glabrata*	Up to 98% eradication (XTT assay)	–	ROS generation, mitochondrial stress, gene downregulation	[80]

Rosemary EO	Camphor (21.97%)	Poland	*P. aeruginosa*	Up to 87.56%	Synergy with 1,8-cineole	Membrane disruption mechanisms	[36]
TEO4 (Carvacrol chemotype)	Carvacrol (44.66%)	Spain	*Streptococcus mutans*	94.31% (2MIC), 70.31% (½MIC)	Synergistic with p-cymene	Carvacrol-p-cymene synergy enhances gene inhibition	[81]
Winter Savory EO (WSEO)	Carvacrol (50.45%), p-Cymene (15.73%)	Serbia	*Yersinia enterocolitica*	Up to 76.42%	Less potent than oregano EO	Dose-dependent but lower efficacy compared to other carvacrol-rich EOs	[10]
Thymus sibthorpii EO	Carvacrol (52.62%)	Greece	Methicillin-sensitive *Staphylococcus aureus* (MSSA), Methicillin-resistant *Staphylococcus aureus* (MRSA), *S. aureus*	93.4–95.8% at ½×MIC–4×MIC	None	High carvacrol and p-cymene disrupt biofilms; potent even at low doses	[30]
Thymbra spicata EO	Carvacrol (54.3%), γ-terpinene (12.4%)	Turkey	*P. aeruginosa, S. aureus, Candida spp.*	Up to 88.13% (*P. aeruginosa*)	Synergism with γ-terpinene	Membrane disruption and QS inhibition	[82]
OEO2 (France)	Carvacrol (59.40%)	France	*Streptococcus mutans* ATCC 700610	89.71% (MIC)	Minor compounds (p-cymene, γ-terpinene)	Superior gene suppression compared to OEO1	[25]
Origanum vulgare	Carvacrol (65.7%)	Hungary	*Neisseria gonorrhoeae, Streptococcus suis*	70% (*S. suis*); increased biomass (*N. gonorrhoeae*)	None	High doses may enhance biofilm in *N. gonorrhoeae*	[31]
Origanum vulgare EO	Carvacrol (78.72%)	Greece	MSSA, MRSA, *S. aureus* ATCC 29213	~93.1–95.8% (½×MIC–4×MIC)	p-Cymene enhances activity	Clear dose-response; dominant antimicrobial component	[30]
Oregano EO (OREO)	Carvacrol (81.00%)	India	*Yersinia enterocolitica*	Up to 78.89%	None	SEM-confirmed cell damage; 2×MIC most effective	[10]
Origanum majorana EO (OMC)	Carvacrol (84%)	Turkey	*Candida spp.*	~83% germ-tube inhibition	p-Cymene (4.8%)	High carvacrol linked to antifungal activity	[29]

Origanum vulgare EO	Carvacrol (86%)	Kazakhstan	*S. mutans, S. aureus, E. coli*	98% (*S. mutans* at 4–10 mg/mL; $p < 0.05$)	None	Plateau effect above 4 mg/mL	[32]
Carvacrol in PLA-based X-NEs	Carvacrol (core)	Not specified	*P. aeruginosa*, MRSA	99.99% CFU reduction (*P. aeruginosa*)	PLA copolymer + TTMA stabilizer	Full nanoemulsion required for efficacy	[2]
Car + bioAgNP	Carvacrol (dominant)	Brazil	Enteroaggregative *E. coli*, KPC-producing *Klebsiella pneumoniae*	99% (EAEC); 66–97% (KPC-Kp)	BioAgNP	Enhanced activity at low doses	[1]
Oregano EO	Carvacrol, Thymol	Brazil	*S. mutans, S. aureus*	90–100% (*S. mutans*); 50–70% (*S. aureus*)	Toothpaste matrix	Membrane permeability modification; dose-dependent activity	[9]
Niosome-loaded Oregano EO	Carvacrol, Thymol	Iran	*Vibrio vulnificus, L. monocytogenes*	2–4× more effective than free EO	Niosomal encapsulation	Enhanced bioavailability; sub-MIC values effective	[83]
OEO + bioAgNP	Carvacrol, Thymol	Brazil	Enteroaggregative *E. coli*, KPC-producing *K. pneumoniae*	88–99% (EAEC); 80–97% (KPC-Kp)	Fungal-synthesized bioAgNP	Synergy outperformed single agents	[1]
Caraway EO	Carvone (63.7%), Sylvestrene (14.8%)	Egypt	*P. aeruginosa*	60–73% inhibition/eradication	None	Attenuates virulence via carvone	[70]
CEON	Caryophyllene (6.89%) + Cinnamaldehyde	USA	Multi-species oral biofilms	Reduced CFUs (4.81 vs. 6.93 control); absorbance (0.29 vs. 1.14 control)	None specified	Cinnamaldehyde disrupts quorum sensing; nanoemulsion enhances delivery	[12]
Cinnamaldehyde + Eugenol blend	Cinnamaldehyde (0.254 mg/cm²)	Not specified	*Listeria, Salmonella*	76.82–89.16%	Synergy with eugenol (FICI 0.24)	Most effective combination in study; disrupts preformed biofilms	[84]
CZ-EO	Cinnamaldehyde (66.1%)	Belgium	*Candida auris* (fluconazole-resistant)	Complete biofilm disintegration at 0.06% vol/vol	Synergy with fluconazole	Sub-MIC concentrations effective in combination	[7]
Cinnamon EO (CIEO)	Cinnamaldehyde (74.93%)	Sri Lanka	*Y. enterocolitica*	Up to 78.59% (Y9 strain)	Dose-dependent	Higher inhibition at lower temperatures	[10]

Cinnamon EO + AgNPs	Cinnamaldehyde (major)	Unspecified	*Streptococcus agalactiae*	$MBIC_{50}$ range = 1/2–32/64 µg/mL	Synergistic for planktonic (FIC < 0.5)	Antagonistic for biofilms in most isolates	[8]
Cinnamon/Cardamom EO	Cinnamaldehyde + Cardamom compounds	Iran	*Bacillus subtilis*, *E. coli*	*B. subtilis*: 98% (CV); *E. coli*: 58% (CV)	Synergy for *B. subtilis* (FICI < 0.5)	Additive/indifferent for *E. coli*	[13]
Lemongrass EO (LEO)	Citral (59.8%)	China	*C. albicans*, *S. aureus*	~80% biomass reduction at 0.3125% LEO	None	Optimal efficacy at low concentration; higher doses ineffective	[59]
Citral (Component)	Citral (59.8%)	N/A	*C. albicans*, *S. aureus*	74–82% biomass reduction; 87–96% viability reduction at 0.5%	None	Downregulates virulence and quorum-sensing genes	[59]
Lemongrass EO (CMLEO)	Citral (83.17%)	Brazil	*C. albicans*	84% biomass reduction (8×MIC)	Chitosan microparticles	Encapsulation improves antifungal efficacy	[58]
Lemongrass EO (CMLEO)	Citral (83.17%)	Brazil	*C. albicans*	~80% inhibition at 2048 µg/mL	Encapsulation	Stronger activity than raw EO	[61]
Citronellol	Citronellol	N/A	*E. coli*	53% metabolic activity reduction	None	Intermediate potency; reduces culturability by 90%	[62]
Cymbopogon citratus Fractions F7/F10	Citronellol (49.3%), γ-Dodecalactone (70.0%)	Cameroon	*C. albicans*	Up to ~66% reduction	None	Fungicidal at MIC; disrupts fungal walls and mitochondria	[60]
Cuminum cyminum EO	Cuminaldehyde (42.4%)	Tunisia	*Vibrio spp.*	Up to 76.29% at 50 mg/mL	None	Molecular docking supports QS inhibition	[73]
Garlic EO (EOG)	Diallyl trisulfide (25.13%)	Spain	*L. monocytogenes*	100% at MIC & 2× MIC	None	Disrupts SH-groups; strong dose-dependence	[11]
Citrus limetta EO (CLEO)	D-Limonene (85.71%)	India	*E. coli*, *P. aeruginosa*, *S. aureus*	90.6–95.6% at 0.5 mg/mL	None	ROS generation and cytotoxicity linked to dose-response	[55]
Cinnamon EO (EOC)	E-cinnamaldehyde (76.54%)	Spain	*L. monocytogenes* CECT 4032	50% at 0.5×MIC; 100% at MIC & 2×MIC (initial biofilms); 61% (preformed)	Dose-dependent ($p < 0.05$)	Cinnamaldehyde disrupts cell envelope	[11]

Cinnamon EO (CiEO)	E-cinnamaldehyde (84.23%)	Iran	*E. coli*, *B. subtilis*	*E. coli*: 100% (CV), 99% (XTT); *B. subtilis*: 55% (CV), 36% (XTT)	None specified	High cinnamaldehyde content linked to protein interaction	[13]
Agastache rugosa EO (AREO)	Estragole (88.69%)	South Korea	*S. mutans*	>90% at 0.04 mg/mL	None	Linked to adhesion reduction (46.2% to 6.2%)	[63]
Rosemary EO (REO)	Eucalyptol (42.1%)	China	*S. aureus*, *E. coli*	82.07% (*S. aureus*), 71.98% (*E. coli*)	–	Disrupts proteins and cell structures	[34]
Cinnamomum verum EO	Eugenol (77.22%)	Sri Lanka	*C. albicans*, *Candida tropicalis*, *Candida dubliniensis*	$MBIC_{50}$: 0.1 mg/mL (*C. albicans*), 0.2 mg/mL (others)	Dose-dependent	Eugenol disrupts membranes and cell walls	[4]
Clove EO (EOC)	Eugenol (78.85%)	Tunisia	*L. monocytogenes*, *Salmonella*	77.2% (*L. monocytogenes* at 2× MIC)	None	Higher efficacy against Gram-positive pathogens; dose-dependent	[76]
CZEO	Eugenol (8.17%) + Cinnamaldehyde	Sri Lanka	*Staphylococcus saprophyticus*, *Staphylococcus pseudintermedius*	Up to 85% (*S. saprophyticus*); 50% (*S. pseudintermedius*)	None specified	4×MIC needed for preformed biofilms; no effect on *S. schleiferi*	[6]
Eugenol Essential Oil (EEO)	Eugenol (96.35%)	Egypt	*Helicobacter pylori*	73.21% at 50 µg/mL	None	Strong dose-dependent inhibition; effective against resistant strains	[85]
Cymbopogon flexuosus EO14	Geranial (41.2%) + Neral (31.0%)	Poland	MRSA	Inhibited biofilm formation at MBIC = 1 mg/mL	None	Ineffective against mature biofilms	[57]
Thymus × citriodorus (TC)	Geraniol (27.5%)	Portugal	*Cutibacterium acnes* (clinical strains)	EC_{50} Biomass: 0.062–0.210%	Thymol (9.2%)	Geraniol dominates activity; disrupts membranes at low doses	[44]
Cymbopogon martinii EO	Geraniol (83.5%)	Germany	*Streptococcus mitis*, *Enterococcus faecalis*	$\Delta log_{10}CFU = 2.75$ (MIC/2)	Comparable to TAP	Strongest activity at MIC/2; effective in irrigation protocols	[50]

Origanum vulgare Essential Oil	Guaiacol–ρ-vinil (68.67%)	France	*S. aureus, E. coli, L. monocytogenes*	10.36–54.05% (*S. aureus*)	Minor terpenes (p-cymene, γ-terpinene)	Activity attributed to guaiacol–ρ-vinil and synergism	[33]
Anethum graveolens EO	Limonene (48.05%), Carvone (37.94%)	France	*Listeria, Vibrio, Shigella*	>50% reduction at 1× MIC	Limonene shows anti-adhesion	Limonene more effective than carvone; QS inhibition observed	[67]
Citrus limon EO (CLEO)	Limonene (60.7%)	Slovakia	*S. enterica*, *Y. enterocolitica*	Up to 93% (*Y. enterocolitica*), ~66% (*S. enterica*)	None	MALDI-TOF confirms protein disruption	[56]
Citrus limon EO	Limonene (64.8%)	Argentina	*N. gonorrhoeae*, *S. suis*	95% (*N. gonorrhoeae*), 40–60% (*S. suis*)	None	Strong bactericidal and membrane-disruptive effects	[31]
Mandarin EO (MEO)	Limonene (78.89%)	China	*S. aureus*, *E. coli*	Significant inhibition of initial adhesion and mature biofilms	Chitosan nanoparticles (1:1 ratio)	Enhanced antibiofilm delivery via nanoparticles	[52]
Citrus reticulata Blanco cv. Tankan Peel EO (CPEO)	Limonene (95.993%)	China	*L. monocytogenes*	EPS reduced: proteins (−47.77%), polysaccharides (−54.74%), eDNA (−63.74%) at 2%	None	Limonene disrupts membranes and inhibits EPS synthesis	[51]
Essential Orange Oil (EOO)	Limonene (not specified)	Egypt	*Bacillus cereus*	52.1% (SS), 55.6% (AF), 65.3% (AL) surfaces at sub-MIC doses	Combined with CFF	Synergy enhances gene regulation (sinR/calY/spo0A)	[54]
Lavender EO	Linalool (35%), Linalyl acetate (35%)	Poland	*Candida spp.*	~75–85%	–	Moderate antifungal activity; tested at MIC	[23]
Lavandula x intermedia EO	Linalool (36.0%), Linalyl acetate (27.3%)	Italy	*Streptococcus agalactiae*, *C. albicans*	Significant reduction ($p < 0.05$)	–	Preserves *Lactobacillus*; better against mature biofilms	[22]
Salvia sclarea EO (SSEO)	Linalool acetate (49.1%), Linalool (20.6%)	Slovakia	*P. fluorescens*	Up to 87.34% (vapor phase)	–	Activity linked to oxygenated monoterpenes	[39]

Citrus aurantium EO (CAEO)	Linalyl acetate (63.37%)	Slovakia	*Stenotrophomonas maltophilia*, *B. subtilis*	50% MBIC at 8.47–8.56 µL/mL	None	Strain-specific; complete biofilm degradation at day 14	[53]
L-EO (Lavender)	Linalyl acetate, linalool	Not specified	*S. aureus*	No eradication	–	Low impact in liquid/vapor forms	[17]
Peppermint EO	Menthol (40%), Menthones (20%)	N/A	*Candida spp.*	75–85% inhibition	None	Dose-dependent EPS disruption	[23]
Mentha arvensis EO	Menthol (73.8%)	Italy	*Streptococcus mitis*, *S. sanguinis*	Moderate to strong reduction	Synergistic combinations	Less effective than *L. x intermedia*	[22]
Etlingera pavieana EO	Methyl chavicol (79.34%)	Thailand	*S. mutans/sobrinus*	97.02% (*S. sobrinus* at 1.6% v/v)	Synergy with trans-anethole	Sub-MIC activity (>90% inhibition)	[86]
Cinnamon Oil (CO)	Not reported	India	*S. aureus* (biofilm-forming)	40% reduction with mupirocin-CO cream	Synergistic with mupirocin	Monoterpenes, sesquiterpenes, phenylpropenes enhance activity	[16]
Origanum vulgare	p-Cymene (13.2%)	Hungary	*N. gonorrhoeae, S. suis*	Increased biomass (*N. gonorrhoeae* at high doses)	Carvacrol (65.7%)	Non-linear dose-response; weak standalone activity	[31]
Thyme EO (Egypt)	p-Cymene (34.25%)	Egypt	MDR *Salmonella typhimurium*	~60% at MIC/2	None	Moderate activity; plateau effect at lower concentrations	[42]
Thyme EO (THEO)	p-Cymene (40.91%)	India	*Y. enterocolitica*	Up to 78.14% (meat matrix); 0.64 log CFU/g reduction	None	Strongest activity in food matrix; thymol contributes	[10]
LEW (Post-Distillation Waste)	Phenolic acid glycosides	Slovenia	*C. jejuni*	Similar to LEF; 0.51 surface coverage at 2× MIC	–	Comparable to LEF; better at lower doses	[24]
Cinnamaldehyde	Pure compound	Unspecified	*L. monocytogenes*, *S. typhimurium*	57.46% (*L. mono*), 51.44% (*S. typhimurium*) (preformed biofilms)	Synergistic with eugenol	Moderate alone; improved efficacy in combination	[84]
β-Caryophyllene	Pure compound	Not specified	*Listeria, Salmonella*	≤28.63%	Additive in blends	Weak standalone activity; better in combination	[84]

LEF (Ethanol Extract)	Rosmarinic acid, ferulic acid	Slovenia	*C. jejuni*	Significant degradation at 8× MIC	–	Phenolic acids support moderate anti-adhesion	[24]
Jatropha gossypifolia EO	Sesquiterpenes (74.86%)	Egypt	*E. coli*	39.25% at 1000 µg/mL	None	Weak dose-response; minimal inhibition at low doses	[87]
Origanum majorana EO	Terpenes (assumed)	Tunisia	MDR clinical strains	~17% (selected strains)	None	Delayed action; low effective concentration	[5]
Sage EO	Thujones (33%), Camphor (20%)	Not specified	*Candida* spp.	~60–70%	–	Weakest activity among tested EOs	[23]
Thymus serpyllum EO (TSEO)	Thymol (18.8%), Carvacrol (17.4%)	Slovakia	*P. aeruginosa*	72.93% inhibition (Crystal Violet assay)	None	Synergistic thymol-carvacrol activity; strong dose-dependent effects	[47]
TEO6 (Thymol chemotype)	Thymol (27.96%)	Spain	*S. mutans*	95.90% (2MIC), 77.60% (½MIC)	p-Cymene enhances membrane interaction	High thymol activity despite lower concentration; synergistic with p-cymene	[81]
Lippia origanoides EO (Thymol chemotype)	Thymol (32.7%), Carvacrol (18.8%)	Colombia	S. Enteritidis	>60% inhibition at 0.13 mg/mL	Synergism with carvacrol	Membrane damage, oxidative stress, and metabolic disruption	[88]
Satureja hortensis EO	Thymol (41.28%), γ-terpinene (37.63%)	Iran	*E. coli*, S. Enteritidis	Up to 26.8% (*E. coli*), significant for *Salmonella*	None noted	Strong antimicrobial effect but moderate antibiofilm activity	[89]
T-EO (Thyme)	Thymol (44%)	Poland	Clinical *S. aureus* (MSSA/MRSA)	57–92% reduction via TTC/live-dead staining	None	High thymol correlates with strong antimicrobial/antibiofilm effects	[17]

Thyme EO (Hungary)	Thymol (52.33–62.46%)	Hungary	*Haemophilus influenzae*, *P. aeruginosa*	72.93% inhibition (*P. aeruginosa*) at MIC/2	None	Dose-dependent; γ-terpinene enhances activity in early flowering stage	[41]
Thymus fallax EO	Thymol (67.75%)	Iran	*Streptococcus* spp., *S. aureus*, *C. albicans*	>95% at 8 µL/mL; >50% at 0.015 µL/mL for some strains	None	High thymol content linked to potent activity; rapid biofilm reduction	[46]
Thyme Oil (China)	Thymol (70.76%)	China	*E. faecalis* (antibiotic-resistant)	Significant reduction at 128–256 µg/mL ($P < 0.05$)	None	Reduces EPS production and motility; disrupts pili/polysaccharide genes	[43]
Thymus vulgaris Essential Oil	Thymol (75.46%)	Egypt	*C. acnes*, *Staphylococcus epidermidis*	Complete inhibition at 0.026 g/mL; eradication at 0.053 g/mL	None	Strong activity due to thymol and caryophyllene; dose-dependent	[49]
Thyme EO (Tunisia)	Thymol (assumed major)	Tunisia	MDR *E. coli*, *K. pneumoniae*, *S. aureus*	Up to 99.51% for *Salmonella*	None	Broad-spectrum activity against MDR strains; anti-quorum sensing effects	[5]
Thy + bioAgNP	Thymol (dominant)	Brazil	Enteroaggregative *E. coli*, KPC-producing *K. pneumoniae*	98–100% (EAEC); 62–100% (KPC-Kp)	Fungal-synthesized bioAgNP	Strongest antibiofilm efficacy in preformed biofilms	[1]
CC-EO	trans-Cinnamaldehyde (85.5%)	China	*Candida auris*	Significant disruption at 0.02% v/v ($p < 0.001$)	Nano-formulation retained activity	No dose-dependency noted	[3]
Onion EO (EOO)	Trisulfide dipropyl (35.46%)	Spain	*L. monocytogenes*	77–100% (0.5×MIC to 2×MIC)	Dose-dependent	Sulfur thiosulfates implicated in inhibition	[11]
Cistus ladanifer EO	α-Pinene (50%)	Portugal	*C. acnes*	EC_{50}: 0.083–>0.500%	None	Limited antibiofilm activity; high doses required	[44]
Cardamom EO (GCEO)	α-Terpinyl acetate (34.95%), 1,8-Cineole (25.30%)	Pakistan	*E. coli, Salmonella*	64.29–100% (dose-dependent)	High synergy between components	Disrupts virulence factors via α-terpinyl acetate	[74]

Sage Oil	α-Thujone (29.7%), Camphor (16.6%)	Serbia	*P. aeruginosa*	84.1–99.6% (pre-adhesion), 81.3–98.7% (mature)	–	High α-thujone/camphor chemotype; inconsistent dose-response	[37]
Sage Oil	α-Thujone (29.7%), Camphor (16.6%)	Serbia	*P. aeruginosa*	84.1–99.6% (pre-adhesion)	–	Effective even at low doses	[37]

Legend

References

1. Scandorieiro, S.; Teixeira, F.M.M.B.; Nogueira, M.C.L.; Panagio, L.A.; de Oliveira, A.G.; Durán, N.; Nakazato, G.; Kobayashi, R.K.T. Antibiofilm Effect of Biogenic Silver Nanoparticles Combined with Oregano Derivatives against Carbapenem-Resistant Klebsiella Pneumoniae. *Antibiotics* **2023**, *12*, 756, doi:10.3390/antibiotics12040756.
2. Oz, Y.; Nabawy, A.; Fedeli, S.; Gupta, A.; Huang, R.; Sanyal, A.; Rotello, V.M. Biodegradable Poly(Lactic Acid) Stabilized Nanoemulsions for the Treatment of Multidrug-Resistant Bacterial Biofilms. *ACS Appl. Mater. Interfaces* **2021**, *13*, 40325–40331, doi:10.1021/acsami.1c11265.
3. Rosato, R.; Napoli, E.; Granata, G.; Di Vito, M.; Garzoli, S.; Geraci, C.; Rizzo, S.; Torelli, R.; Sanguinetti, M.; Bugli, F. Study of the Chemical Profile and Anti-Fungal Activity against Candida Auris of Cinnamomum Cassia Essential Oil and of Its Nano-Formulations Based on Polycaprolactone. *Plants* **2023**, *12*, 358, doi:10.3390/plants12020358.
4. Wijesinghe, G.K.; de Oliveira, T.R.; Maia, F.C.; de Feiria, S.B.; Barbosa, J.P.; Joia, F.; Boni, G.C.; Höfling, J.F. Efficacy of True Cinnamon (Cinnamomum Verum) Leaf Essential Oil as a Therapeutic Alternative for Candida Biofilm Infections. *Iran. J. Basic Med. Sci.* **2021**, *24*, 787–795, doi:10.22038/ijbms.2021.53981.12138.
5. Alibi, S.; Ben Selma, W.; Ramos-Vivas, J.; Smach, M.A.; Touati, R.; Boukadida, J.; Navas, J.; Ben Mansour, H. Anti-Oxidant, Antibacterial, Anti-Biofilm, and Anti-Quorum Sensing Activities of Four Essential Oils against Multidrug-Resistant Bacterial Clinical Isolates. *Curr. Res. Transl. Med.* **2020**, *68*, 59–66, doi:10.1016/j.retram.2020.01.001.
6. Albuquerque, V. de Q.; Soares, M.J.C.; Matos, M.N.C.; Cavalcante, R.M.B.; Guerrero, J.A.P.; Soares Rodrigues, T.H.; Gomes, G.A.; de Medeiros Guedes, R.F.; Castelo-Branco, D. de S.C.M.; Goes da Silva, I.N.; et al. Anti-Staphylococcal Activity of Cinnamomum Zeylanicum Essential Oil against Planktonic and Biofilm Cells Isolated from Canine Otological Infections. *Antibiot. Basel Switz.* **2021**, *11*, 4, doi:10.3390/antibiotics11010004.
7. Di Vito, M.; Garzoli, S.; Rosato, R.; Mariotti, M.; Gervasoni, J.; Santucci, L.; Ovidi, E.; Cacaci, M.; Lombarini, G.; Torelli, R.; et al. A New Potential Resource in the Fight against Candida Auris: The Cinnamomum Zeylanicum Essential Oil in Synergy with Antifungal Drug. *Microbiol. Spectr.* **2023**, *11*, e04385-22, doi:10.1128/spectrum.04385-22.
8. Abd El-Aziz, N.K.; Ammar, A.M.; El-Naenaeey, E.Y.M.; El Damaty, H.M.; Elazazy, A.A.; Hefny, A.A.; Shaker, A.; Eldesoukey, I.E. Antimicrobial and Antibiofilm Potentials of Cinnamon Oil and Silver Nanoparticles against Streptococcus Agalactiae Isolated from Bovine Mastitis: New Avenues for Countering Resistance. *BMC Vet. Res.* **2021**, *17*, 136, doi:10.1186/s12917-021-02842-9.
9. de Oliveira Carvalho, I.; Purgato, G.A.; Píccolo, M.S.; Pizziolo, V.R.; Coelho, R.R.; Diaz-Muñoz, G.; Alves Nogueira Diaz, M. *In vitro* Anticariogenic and Antibiofilm Activities of Toothpastes Formulated with Essential Oils. *Arch. Oral Biol.* **2020**, *117*, 104834, doi:10.1016/j.archoralbio.2020.104834.
10. Vidaković Knežević, S.; Knežević, S.; Vranešević, J.; Milanov, D.; Ružić, Z.; Karabasil, N.; Kocić-Tanackov, S. Using Essential Oils to Reduce Yersinia Enterocolitica in Minced Meat and in Biofilms. *Foods* **2024**, *13*, 806, doi:10.3390/foods13050806.
11. Somrani, M.; Inglés, M.-C.; Debbabi, H.; Abidi, F.; Palop, A. Garlic, Onion, and Cinnamon Essential Oil Anti-Biofilms' Effect against Listeria Monocytogenes. *Foods* **2020**, *9*, 567, doi:10.3390/foods9050567.
12. Jeong, Y.-J.; Kim, H.-E.; Han, S.-J.; Choi, J.-S. Antibacterial and Antibiofilm Activities of Cinnamon Essential Oil Nanoemulsion against Multi-Species Oral Biofilms. *Sci. Rep.* **2021**, *11*, 5911, doi:10.1038/s41598-021-85375-3.
13. Pourkhosravani, E.; Dehghan Nayeri, F.; Mohammadi Bazargani, M. Decoding Antibacterial and Antibiofilm Properties of Cinnamon and Cardamom Essential Oils: A Combined Molecular Docking and Experimental Study. *AMB Express* **2021**, *11*, 143, doi:10.1186/s13568-021-01305-6.

14. Tartor, Y.H.; Elmowalid, G.A.; Hassan, M.N.; Shaker, A.; Ashour, D.F.; Saber, T. Promising Anti-Biofilm Agents and Phagocytes Enhancers for the Treatment of Candida Albicans Biofilm–Associated Infections. *Front. Cell. Infect. Microbiol.* **2022**, *12*, doi:10.3389/fcimb.2022.807218.
15. El-Baz, A.M.; Mosbah, R.A.; Goda, R.M.; Mansour, B.; Sultana, T.; Dahms, T.E.S.; El-Ganiny, A.M. Back to Nature: Combating Candida Albicans Biofilm, Phospholipase and Hemolysin Using Plant Essential Oils. *Antibiotics* **2021**, *10*, 81, doi:10.3390/antibiotics10010081.
16. Sundaramoorthy, M.; Karuppaiah, A.; Nithyanth, M.; Baberoselin, R.; Ramesh, S.; Geetha, N.; Veintramuthu, S. Formulation Development of Cream with Mupirocin and Essential Oils for Eradication of Biofilm Mediated Antimicrobial Resistance. *Arch. Microbiol.* **2021**, *203*, 1707–1715, doi:10.1007/s00203-020-02175-5.
17. Brożyna, M.; Paleczny, J.; Kozłowska, W.; Chodaczek, G.; Dudek-Wicher, R.; Felińczak, A.; Gołębiewska, J.; Górniak, A.; Junka, A. The Antimicrobial and Antibiofilm *In vitro* Activity of Liquid and Vapour Phases of Selected Essential Oils against Staphylococcus Aureus. *Pathogens* **2021**, *10*, 1207, doi:10.3390/pathogens10091207.
18. Cai, K.; Liu, Y.; Yue, Y.; Liu, Y.; Guo, F. Essential Oil Nanoemulsion Hydrogel with Anti-Biofilm Activity for the Treatment of Infected Wounds. *Polymers* **2023**, *15*, 1376, doi:10.3390/polym15061376.
19. Gherasim, O.; Popescu, R.C.; Grumezescu, V.; Mogoşanu, G.D.; Mogoantă, L.; Iordache, F.; Holban, A.M.; Vasile, B. Ştefan; Bîrcă, A.C.; Oprea, O.-C.; et al. MAPLE Coatings Embedded with Essential Oil-Conjugated Magnetite for Anti-Biofilm Applications. *Materials* **2021**, *14*, 1612, doi:10.3390/ma14071612.
20. Iseppi, R.; Mariani, M.; Benvenuti, S.; Truzzi, E.; Messi, P. Effects of Melaleuca Alternifolia Chell (Tea Tree) and Eucalyptus Globulus Labill. Essential Oils on Antibiotic-Resistant Bacterial Biofilms. *Molecules* **2023**, *28*, 1671, doi:10.3390/molecules28041671.
21. Fadel, B.A.; Elwakil, B.H.; Fawzy, E.E.; Shaaban, M.M.; Olama, Z.A. Nanoemulsion of Lavandula Angustifolia Essential Oil/Gold Nanoparticles: Antibacterial Effect against Multidrug-Resistant Wound-Causing Bacteria. *Molecules* **2023**, *28*, 6988, doi:10.3390/molecules28196988.
22. Iseppi, R.; Tardugno, R.; Brighenti, V.; Benvenuti, S.; Sabia, C.; Pellati, F.; Messi, P. Phytochemical Composition and *In vitro* Antimicrobial Activity of Essential Oils from the Lamiaceae Family against Streptococcus Agalactiae and Candida Albicans Biofilms. *Antibiot. Basel Switz.* **2020**, *9*, 592, doi:10.3390/antibiotics9090592.
23. Karpiński, T.M.; Ożarowski, M.; Seremak-Mrozikiewicz, A.; Wolski, H. Anti-Candida and Antibiofilm Activity of Selected Lamiaceae Essential Oils. *Front. Biosci. Landmark Ed.* **2023**, *28*, 28, doi:10.31083/j.fbl2802028.
24. Ramić, D.; Bucar, F.; Kunej, U.; Dogša, I.; Klančnik, A.; Smole Možina, S. Antibiofilm Potential of Lavandula Preparations against Campylobacter Jejuni. *Appl. Environ. Microbiol.* **2021**, *87*, e01099-21, doi:10.1128/AEM.01099-21.
25. Yuan, Y.; Sun, J.; Song, Y.; Raka, R.N.; Xiang, J.; Wu, H.; Xiao, J.; Jin, J.; Hui, X. Antibacterial Activity of Oregano Essential Oils against Streptococcus Mutans *In vitro* and Analysis of Active Components. *BMC Complement. Med. Ther.* **2023**, *23*, 61, doi:10.1186/s12906-023-03890-4.
26. Naccari, C.; Ginestra, G.; Micale, N.; Palma, E.; Galletta, B.; Costa, R.; Vadalà, R.; Nostro, A.; Cristani, M. Binary Combinations of Essential Oils: Antibacterial Activity Against Staphylococcus Aureus, and Antioxidant and Anti-Inflammatory Properties. *Molecules* **2025**, *30*, 438, doi:10.3390/molecules30030438.
27. Kapustová, M.; Puškárová, A.; Bučková, M.; Granata, G.; Napoli, E.; Annušová, A.; Mesárošová, M.; Kozics, K.; Pangallo, D.; Geraci, C. Biofilm Inhibition by Biocompatible Poly(ε-Caprolactone) Nanocapsules Loaded with Essential Oils and Their Cyto/Genotoxicity to Human Keratinocyte Cell Line. *Int. J. Pharm.* **2021**, *606*, 120846, doi:10.1016/j.ijpharm.2021.120846.

28. Zhan, X.; Tan, Y.; Lv, Y.; Fang, J.; Zhou, Y.; Gao, X.; Zhu, H.; Shi, C. The Antimicrobial and Antibiofilm Activity of Oregano Essential Oil against Enterococcus Faecalis and Its Application in Chicken Breast. *Foods* **2022**, *11*, 2296, doi:10.3390/foods11152296.
29. Kaskatepe, B.; Aslan Erdem, S.; Ozturk, S.; Safi Oz, Z.; Subasi, E.; Koyuncu, M.; Vlainić, J.; Kosalec, I. Antifungal and Anti-Virulent Activity of Origanum Majorana L. Essential Oil on Candida Albicans and In Vivo Toxicity in the Galleria Mellonella Larval Model. *Mol. Basel Switz.* **2022**, *27*, 663, doi:10.3390/molecules27030663.
30. Ersanli, C.; Tzora, A.; Skoufos, I.; Fotou, K.; Maloupa, E.; Grigoriadou, K.; Voidarou, C. (Chrysa); Zeugolis, D.I. The Assessment of Antimicrobial and Anti-Biofilm Activity of Essential Oils against Staphylococcus Aureus Strains. *Antibiotics* **2023**, *12*, 384, doi:10.3390/antibiotics12020384.
31. Jurado, P.; Uruén, C.; Martínez, S.; Lain, E.; Sánchez, S.; Rezusta, A.; López, V.; Arenas, J. Essential Oils of Pinus Sylvestris, Citrus Limon and Origanum Vulgare Exhibit High Bactericidal and Anti-Biofilm Activities against Neisseria Gonorrhoeae and Streptococcus Suis. *Biomed. Pharmacother. Biomedecine Pharmacother.* **2023**, *168*, 115703, doi:10.1016/j.biopha.2023.115703.
32. Badekova, K.Z.; Atazhanova, G.A.; Kacergius, T.; Akhmetova, S.B.; Smagulov, M.K. Formulation of an Origanum Vulgare Based Dental Gel with Antimicrobial Activity. *J. Taibah Univ. Med. Sci.* **2021**, *16*, 712–718, doi:10.1016/j.jtumed.2021.05.009.
33. Merghni, A.; Haddaji, N.; Bouali, N.; Alabbosh, K.F.; Adnan, M.; Snoussi, M.; Noumi, E. Comparative Study of Antibacterial, Antibiofilm, Antiswarming and Antiquorum Sensing Activities of Origanum Vulgare Essential Oil and Terpinene-4-Ol against Pathogenic Bacteria. *Life Basel Switz.* **2022**, *12*, 1616, doi:10.3390/life12101616.
34. Liu, T.; Wang, J.; Gong, X.; Wu, X.; Liu, L.; Chi, F. Rosemary and Tea Tree Essential Oils Exert Antibiofilm Activities *In vitro* against Staphylococcus Aureus and Escherichia Coli. *J. Food Prot.* **2020**, *83*, 1261–1267, doi:10.4315/0362-028X.JFP-19-337.
35. Kabotso, D.E.K.; Neglo, D.; Gaba, S.E.; Danyo, E.K.; Dayie, A.D.; Asantewaa, A.A.; Kotey, F.C.N.; Dayie, N.T.K.D. *In vitro* Evaluation of Rosemary Essential Oil: GC-MS Profiling, Antibacterial Synergy, and Biofilm Inhibition. *Pharmaceuticals* **2024**, *17*, 1653, doi:10.3390/ph17121653.
36. Brożyna, M.; Paleczny, J.; Kozłowska, W.; Ciecholewska-Juśko, D.; Parfieńczyk, A.; Chodaczek, G.; Junka, A. Chemical Composition and Antibacterial Activity of Liquid and Volatile Phase of Essential Oils against Planktonic and Biofilm-Forming Cells of Pseudomonas Aeruginosa. *Molecules* **2022**, *27*, 4096, doi:10.3390/molecules27134096.
37. Pejčić, M.; Stojanović-Radić, Z.; Genčić, M.; Dimitrijević, M.; Radulović, N. Anti-Virulence Potential of Basil and Sage Essential Oils: Inhibition of Biofilm Formation, Motility and Pyocyanin Production of Pseudomonas Aeruginosa Isolates. *Food Chem. Toxicol. Int. J. Publ. Br. Ind. Biol. Res. Assoc.* **2020**, *141*, 111431, doi:10.1016/j.fct.2020.111431.
38. Selim, S.; Almuhayawi, M.S.; Alqhtani, H.; Al Jaouni, S.K.; Saleh, F.M.; Warrad, M.; Hagagy, N. Anti-*Salmonella* and Antibiofilm Potency of Salvia Officinalis L. Essential Oil against Antibiotic-Resistant *Salmonella* Enterica. *Antibiot. Basel Switz.* **2022**, *11*, 489, doi:10.3390/antibiotics11040489.
39. Kačániová, M.; Vukovic, N.L.; Čmiková, N.; Galovičová, L.; Schwarzová, M.; Šimora, V.; Kowalczewski, P.Ł.; Kluz, M.I.; Puchalski, C.; Bakay, L.; et al. Salvia Sclarea Essential Oil Chemical Composition and Biological Activities. *Int. J. Mol. Sci.* **2023**, *24*, 5179, doi:10.3390/ijms24065179.
40. Sateriale, D.; Forgione, G.; De Cristofaro, G.A.; Pagliuca, C.; Colicchio, R.; Salvatore, P.; Paolucci, M.; Pagliarulo, C. Antibacterial and Antibiofilm Efficacy of Thyme (Thymus Vulgaris L.) Essential Oil against Foodborne Illness Pathogens, *Salmonella* Enterica Subsp. Enterica Serovar Typhimurium and Bacillus Cereus. *Antibiotics* **2023**, *12*, 485, doi:10.3390/antibiotics12030485.

41. Bakó, C.; Balázs, V.L.; Kerekes, E.; Kocsis, B.; Nagy, D.U.; Szabó, P.; Micalizzi, G.; Mondello, L.; Krisch, J.; Pethő, D.; et al. Flowering Phenophases Influence the Antibacterial and Anti-Biofilm Effects of Thymus Vulgaris L. Essential Oil. *BMC Complement. Med. Ther.* **2023**, *23*, 168, doi:10.1186/s12906-023-03966-1.

42. Morshdy, A.E.M.A.; El-tahlawy, A.S.; Qari, S.H.; Qumsani, A.T.; Bay, D.H.; Sami, R.; Althubaiti, E.H.; Mansour, A.M.A.; Aljahani, A.H.; Hafez, A.E.-S.E.; et al. Anti-Biofilms' Activity of Garlic and Thyme Essential Oils against *Salmonella* Typhimurium. *Molecules* **2022**, *27*, 2182, doi:10.3390/molecules27072182.

43. Liu, F.; Jin, P.; Gong, H.; Sun, Z.; Du, L.; Wang, D. Antibacterial and Antibiofilm Activities of Thyme Oil against Foodborne Multiple Antibiotics-Resistant Enterococcus Faecalis. *Poult. Sci.* **2020**, *99*, 5127–5136, doi:10.1016/j.psj.2020.06.067.

44. Oliveira, A.S.; Gaspar, C.; Rolo, J.; Palmeira-de-Oliveira, R.; Teixeira, J.P.; Martinez-de-Oliveira, J.; Palmeira-de-Oliveira, A. Comparative Efficacy of Essential Oils against Cutibacterium Acnes: Effect upon Strains from Phylotypes with Different Virulence Patterns. *Microb. Pathog.* **2025**, *199*, 107159, doi:10.1016/j.micpath.2024.107159.

45. Maniki, E.; Kostoglou, D.; Paterakis, N.; Nikolaou, A.; Kourkoutas, Y.; Papachristoforou, A.; Giaouris, E. Chemical Composition, Antioxidant, and Antibiofilm Properties of Essential Oil from Thymus Capitatus Plants Organically Cultured on the Greek Island of Lemnos. *Mol. Basel Switz.* **2023**, *28*, 1154, doi:10.3390/molecules28031154.

46. Moshaverinia, M.; Sahmeddini, S.; Lavaee, F.; Zareshahrabadi, Z.; Zomorodian, K. 10.1016/j.Psj.2020.06.067. *BioMed Res. Int.* **2022**, *2022*, 9744153, doi:10.1155/2022/9744153.

47. Kačániová, M.; Garzoli, S.; Ben Hsouna, A.; Bianchi, A.; Kluz, M.I.; Elizondo-Luevano, J.H.; Ban, Z.; Ben Saad, R.; Mnif, W.; Haščík, P. The Potential of Thymus Serpyllum Essential Oil as an Antibacterial Agent against Pseudomonas Aeruginosa in the Preservation of Sous Vide Red Deer Meat. *Foods* **2024**, *13*, 3107, doi:10.3390/foods13193107.

48. Jafri, H.; Ahmad, I. Thymus Vulgaris Essential Oil and Thymol Inhibit Biofilms and Interact Synergistically with Antifungal Drugs against Drug Resistant Strains of Candida Albicans and Candida Tropicalis. *J. Mycol. Medicale* **2020**, *30*, 100911, doi:10.1016/j.mycmed.2019.100911.

49. Abdelhamed, F.M.; Abdeltawab, N.F.; ElRakaiby, M.T.; Shamma, R.N.; Moneib, N.A. Antibacterial and Anti-Inflammatory Activities of Thymus Vulgaris Essential Oil Nanoemulsion on Acne Vulgaris. *Microorganisms* **2022**, *10*, 1874, doi:10.3390/microorganisms10091874.

50. Marinković, J.; Ćulafić, D.M.; Nikolić, B.; Đukanović, S.; Marković, T.; Tasić, G.; Ćirić, A.; Marković, D. Antimicrobial Potential of Irrigants Based on Essential Oils of Cymbopogon Martinii and Thymus Zygis towards *In vitro* Multispecies Biofilm Cultured in Ex Vivo Root Canals. *Arch. Oral Biol.* **2020**, *117*, 104842, doi:10.1016/j.archoralbio.2020.104842.

51. Peng, J.; Chen, G.; Guo, S.; Lin, Z.; Zeng, Y.; Ren, J.; Wang, Q.; Yang, W.; Liang, Y.; Li, J. Anti-Bacterial and Anti-Biofilm Activities of Essential Oil from Citrus Reticulata Blanco Cv. Tankan Peel Against Listeria Monocytogenes. *Foods* **2024**, *13*, 3841, doi:10.3390/foods13233841.

52. Song, X.; Wang, L.; Liu, T.; Liu, Y.; Wu, X.; Liu, L. Mandarin (Citrus Reticulata L.) Essential Oil Incorporated into Chitosan Nanoparticles: Characterization, Anti-Biofilm Properties and Application in Pork Preservation. *Int. J. Biol. Macromol.* **2021**, *185*, 620–628, doi:10.1016/j.ijbiomac.2021.06.195.

53. Kačániová, M.; Terentjeva, M.; Galovičová, L.; Ivanišová, E.; Štefániková, J.; Valková, V.; Borotová, P.; Kowalczewski, P.Ł.; Kunová, S.; Felšöciová, S.; et al. Biological Activity and Antibiofilm Molecular Profile of Citrus Aurantium Essential Oil and Its Application in a Food Model. *Molecules* **2020**, *25*, 3956, doi:10.3390/molecules25173956.

54. Ammar, H.A.; Samy, R.; Reda, F.M.; Hassanein, W.A. Essential Oils and Lactobacillus Metabolites as Alternative Antibiofilm Agents against Foodborne Bacteria and Molecular Analysis of Biofilm Regulatory Genes. *Sci. Rep.* **2025**, *15*, 7576, doi:10.1038/s41598-025-89998-8.

55. Narayanankutty, A.; Visakh, N.U.; Sasidharan, A.; Pathrose, B.; Olatunji, O.J.; Al-Ansari, A.; Alfarhan, A.; Ramesh, V. Chemical Composition, Antioxidant, Anti-Bacterial, and Anti-Cancer Activities of Essential Oils Extracted from Citrus Limetta Risso Peel Waste Remains after Commercial Use. *Molecules* **2022**, *27*, 8329, doi:10.3390/molecules27238329.
56. Kačániová, M.; Čmiková, N.; Vukovic, N.L.; Verešová, A.; Bianchi, A.; Garzoli, S.; Ben Saad, R.; Ben Hsouna, A.; Ban, Z.; Vukic, M.D. Citrus Limon Essential Oil: Chemical Composition and Selected Biological Properties Focusing on the Antimicrobial (*In vitro* , In Situ), Antibiofilm, Insecticidal Activity and Preservative Effect against *Salmonella* Enterica Inoculated in Carrot. *Plants* **2024**, *13*, 524, doi:10.3390/plants13040524.
57. Piasecki, B.; Biernasiuk, A.; Skiba, A.; Skalicka-Woźniak, K.; Ludwiczuk, A. Composition, Anti-MRSA Activity and Toxicity of Essential Oils from Cymbopogon Species. *Molecules* **2021**, *26*, 7542, doi:10.3390/molecules26247542.
58. Garcia, L.G.S.; da Rocha, M.G.; Lima, L.R.; Cunha, A.P.; de Oliveira, J.S.; de Andrade, A.R.C.; Ricardo, N.M.P.S.; Pereira-Neto, W.A.; Sidrim, J.J.C.; Rocha, M.F.G.; et al. Essential Oils Encapsulated in Chitosan Microparticles against *Candida Albicans* Biofilms. *Int. J. Biol. Macromol.* **2021**, *166*, 621–632, doi:10.1016/j.ijbiomac.2020.10.220.
59. Gao, S.; Liu, G.; Li, J.; Chen, J.; Li, L.; Li, Z.; Zhang, X.; Zhang, S.; Thorne, R.F.; Zhang, S. Antimicrobial Activity of Lemongrass Essential Oil (Cymbopogon Flexuosus) and Its Active Component Citral Against Dual-Species Biofilms of Staphylococcus Aureus and Candida Species. *Front. Cell. Infect. Microbiol.* **2020**, *10*, 603858, doi:10.3389/fcimb.2020.603858.
60. Ngo-Mback, M.N.L.; Babii, C.; Jazet Dongmo, P.M.; Kouipou Toghueo, M.R.; Stefan, M.; Fekam Boyom, F. Anticandidal and Synergistic Effect of Essential Oil Fractions from Three Aromatic Plants Used in Cameroon. *J. Mycol. Médicale* **2020**, *30*, 100940, doi:10.1016/j.mycmed.2020.100940.
61. Garcia, L.G.S.; Rocha, M.G. da; Freire, R.S.; Nunes, P.I.G.; Nunes, J.V.S.; Fernandes, M.R.; Pereira-Neto, W.A.; Sidrim, J.J.C.; Santos, F.A.; Rocha, M.F.G.; et al. Chitosan Microparticles Loaded with Essential Oils Inhibit Duo-Biofilms of Candida Albicans and Streptococcus Mutans. *J. Appl. Oral Sci. Rev. FOB* **2023**, *31*, e20230146, doi:10.1590/1678-7757-2023-0146.
62. Olszewska, M.A.; Gędas, A.; Simões, M. The Effects of Eugenol, Trans-Cinnamaldehyde, Citronellol, and Terpineol on Escherichia Coli Biofilm Control as Assessed by Culture-Dependent and -Independent Methods. *Molecules* **2020**, *25*, 2641, doi:10.3390/molecules25112641.
63. Kim, E.S.; Park, B.-I.; Kim, Y.-H.; Kang, J.; You, Y.-O. The Inhibitory Effect of Agastache Rugosa Essential Oil on the Dental Biofilm. *Molecules* **2024**, *29*, 4907, doi:10.3390/molecules29204907.
64. Khammassi, M.; Polito, F.; Caputo, L.; Abidi, A.; Mabrouk, Y.; Nazzaro, F.; Fratianni, F.; Anouar, E.H.; Snoussi, M.; Noumi, E.; et al. Antibacterial, Antibiofilm, and Chemical Profiles of Ammi Visnaga L. and Foeniculum Vulgare Mill. Essential Oils, and ADMET, Molecular Docking Investigation of Essential Oils Major Components. *Fitoterapia* **2024**, *177*, 106047, doi:10.1016/j.fitote.2024.106047.
65. Tang, C.; Chen, J.; Zhang, L.; Zhang, R.; Zhang, S.; Ye, S.; Zhao, Z.; Yang, D. Exploring the Antibacterial Mechanism of Essential Oils by Membrane Permeability, Apoptosis and Biofilm Formation Combination with Proteomics Analysis against Methicillin-Resistant Staphylococcus Aureus. *Int. J. Med. Microbiol. IJMM* **2020**, *310*, 151435, doi:10.1016/j.ijmm.2020.151435.
66. Abdullah; Algburi, A.; Asghar, A.; Huang, Q.; Mustfa, W.; Javed, H.U.; Zehm, S.; Chikindas, M.L. Black Cardamom Essential Oil Prevents Escherichia Coli O157:H7 and *Salmonella* Typhimurium JSG 1748 Biofilm Formation through Inhibition of Quorum Sensing. *J. Food Sci. Technol.* **2021**, *58*, 3183–3191, doi:10.1007/s13197-020-04821-8.
67. Noumi, E.; Ahmad, I.; Adnan, M.; Merghni, A.; Patel, H.; Haddaji, N.; Bouali, N.; Alabbosh, K.F.; Ghannay, S.; Aouadi, K.; et al. GC/MS Profiling, Antibacterial, Anti-Quorum Sensing, and Antibiofilm Properties of Anethum Graveolens L. Essential Oil: Molecular Docking Study and In-Silico ADME Profiling. *Plants* **2023**, *12*, 1997, doi:10.3390/plants12101997.

68. Mohammadi Pelarti, S.; Karimi Zarehshuran, L.; Babaeekhou, L.; Ghane, M. Antibacterial, Anti-Biofilm and Anti-Quorum Sensing Activities of Artemisia Dracunculus Essential Oil (EO): A Study against *Salmonella* Enterica Serovar Typhimurium and Staphylococcus Aureus. *Arch. Microbiol.* **2021**, *203*, 1529–1537, doi:10.1007/s00203-020-02138-w.

69. Lim, A.C.; Tang, S.G.H.; Zin, N.M.; Maisarah, A.M.; Ariffin, I.A.; Ker, P.J.; Mahlia, T.M.I. Chemical Composition, Antioxidant, Antibacterial, and Antibiofilm Activities of Backhousia Citriodora Essential Oil. *Molecules* **2022**, *27*, 4895, doi:10.3390/molecules27154895.

70. Fekry, M.; Yahya, G.; Osman, A.; Al-Rabia, M.W.; Mostafa, I.; Abbas, H.A. GC-MS Analysis and Microbiological Evaluation of Caraway Essential Oil as a Virulence Attenuating Agent against Pseudomonas Aeruginosa. *Molecules* **2022**, *27*, 8532, doi:10.3390/molecules27238532.

71. Kačániová, M.; Galovičová, L.; Valková, V.; Ďuranová, H.; Štefániková, J.; Čmiková, N.; Vukic, M.; Vukovic, N.L.; Kowalczewski, P.Ł. Chemical Composition, Antioxidant, *In vitro* and In Situ Antimicrobial, Antibiofilm, and Anti-Insect Activity of Cedar Atlantica Essential Oil. *Plants* **2022**, *11*, 358, doi:10.3390/plants11030358.

72. Dudek-Wicher, R.; Junka, A.F.; Migdał, P.; Korzeniowska-Kowal, A.; Wzorek, A.; Bartoszewicz, M. The Antibiofilm Activity of Selected Substances Used in Oral Health Prophylaxis. *BMC Oral Health* **2022**, *22*, 509, doi:10.1186/s12903-022-02532-4.

73. Ghannay, S.; Aouadi, K.; Kadri, A.; Snoussi, M. *In vitro* and In Silico Screening of Anti-Vibrio Spp., Antibiofilm, Antioxidant and Anti-Quorum Sensing Activities of Cuminum Cyminum L. Volatile Oil. *Plants* **2022**, *11*, 2236, doi:10.3390/plants11172236.

74. Abdullah; Asghar, A.; Algburi, A.; Huang, Q.; Ahmad, T.; Zhong, H.; Javed, H.U.; Ermakov, A.M.; Chikindas, M.L. Anti-Biofilm Potential of Elletaria Cardamomum Essential Oil Against Escherichia Coli O157:H7 and *Salmonella* Typhimurium JSG 1748. *Front. Microbiol.* **2021**, *12*, doi:10.3389/fmicb.2021.620227.

75. Noumi, E.; Ahmad, I.; Adnan, M.; Patel, H.; Merghni, A.; Haddaji, N.; Bouali, N.; Alabbosh, K.F.; Kadri, A.; Caputo, L.; et al. Illicium Verum L. (Star Anise) Essential Oil: GC/MS Profile, Molecular Docking Study, In Silico ADME Profiling, Quorum Sensing, and Biofilm-Inhibiting Effect on Foodborne Bacteria. *Molecules* **2023**, *28*, 7691, doi:10.3390/molecules28237691.

76. Somrani, M.; Debbabi, H.; Palop, A. Antibacterial and Antibiofilm Activity of Essential Oil of Clove against Listeria Monocytogenes and *Salmonella* Enteritidis. *Food Sci. Technol. Int. Cienc. Tecnol. Los Aliment. Int.* **2022**, *28*, 331–339, doi:10.1177/10820132211013273.

77. dos Santos, E.A.R.; Tadielo, L.E.; Schmiedt, J.A.; Possebon, F.S.; Pereira, M.O.; Pereira, J.G.; dos Santos Bersot, L. Effect of Ginger Essential Oil and 6-Gingerol on a Multispecies Biofilm of Listeria Monocytogenes, *Salmonella* Typhimurium, and Pseudomonas Aeruginosa. *Braz. J. Microbiol.* **2023**, *54*, 3041–3049, doi:10.1007/s42770-023-01075-2.

78. Zhu, W.; Liu, J.; Zou, Y.; Li, S.; Zhao, D.; Wang, H.; Xia, X. Anti-Biofilm Activity of Laurel Essential Oil against Vibrio Parahaemolyticus. *Foods* **2023**, *12*, 3658, doi:10.3390/foods12193658.

79. Ellboudy, N.M.; Elwakil, B.H.; Shaaban, M.M.; Olama, Z.A. Cinnamon Oil-Loaded Nanoliposomes with Potent Antibacterial and Antibiofilm Activities. *Mol. Basel Switz.* **2023**, *28*, 4492, doi:10.3390/molecules28114492.

80. Gupta, P.; Pruthi, V.; Poluri, K.M. Mechanistic Insights into Candida Biofilm Eradication Potential of Eucalyptol. *J. Appl. Microbiol.* **2021**, *131*, 105–123, doi:10.1111/jam.14940.

81. Park, S.-Y.; Raka, R.N.; Hui, X.-L.; Song, Y.; Sun, J.-L.; Xiang, J.; Wang, J.; Jin, J.-M.; Li, X.-K.; Xiao, J.-S.; et al. Six Spain Thymus Essential Oils Composition Analysis and Their *In vitro* and in Silico Study against Streptococcus Mutans. *BMC Complement. Med. Ther.* **2023**, *23*, 106, doi:10.1186/s12906-023-03928-7.

82. Barak, T.H.; Eryilmaz, M.; Karaca, B.; Servi, H.; Kara Ertekin, S.; Dinc, M.; Ustuner, H. Antimicrobial, Anti-Biofilm, Anti-Quorum Sensing and Cytotoxic Activities of Thymbra Spicata L. Subsp. Spicata Essential Oils. *Antibiotics* **2025**, *14*, 181, doi:10.3390/antibiotics14020181.

83. Sirati, R.; Khajehrahimi, A.E.; Kazempoor, R.; Kakoolaki, S.; Ghorbanzadeh, A. Development, Physicochemical Characterization, and Antimicrobial Evaluation of Niosome-Loaded Oregano Essential Oil against Fish-Borne Pathogens. *Heliyon* **2024**, *10*, e26486, doi:10.1016/j.heliyon.2024.e26486.
84. Purkait, S.; Bhattacharya, A.; Bag, A.; Chattopadhyay, R.R. Evaluation of Antibiofilm Efficacy of Essential Oil Components β-Caryophyllene, Cinnamaldehyde and Eugenol Alone and in Combination against Biofilm Formation and Preformed Biofilms of Listeria Monocytogenes and *Salmonella* Typhimurium. *Lett. Appl. Microbiol.* **2020**, *71*, 195–202, doi:10.1111/lam.13308.
85. Elbestawy, M.K.M.; El-Sherbiny, G.M.; Moghannem, S.A. Antibacterial, Antibiofilm and Anti-Inflammatory Activities of Eugenol Clove Essential Oil against Resistant Helicobacter Pylori. *Molecules* **2023**, *28*, 2448, doi:10.3390/molecules28062448.
86. Wongsariya, K.; Lapirattanakul, J.; Chewchinda, S.; Kanchanadumkerng, P. Anti-Oral Streptococci and Anti-Biofilm Properties of Etlingera Pavieana Essential Oil and Its Bioactive Compounds Proposed for an Alternative Herbal Mouthwash. *Heliyon* **2024**, *10*, e31136, doi:10.1016/j.heliyon.2024.e31136.
87. Gamal El-Din, M.I.; Youssef, F.S.; Altyar, A.E.; Ashour, M.L. GC/MS Analyses of the Essential Oils Obtained from Different Jatropha Species, Their Discrimination Using Chemometric Analysis and Assessment of Their Antibacterial and Anti-Biofilm Activities. *Plants* **2022**, *11*, 1268, doi:10.3390/plants11091268.
88. Guillín, Y.; Cáceres, M.; Stashenko, E.E.; Hidalgo, W.; Ortiz, C. Untargeted Metabolomics for Unraveling the Metabolic Changes in Planktonic and Sessile Cells of *Salmonella* Enteritidis ATCC 13076 after Treatment with Lippia Origanoides Essential Oil. *Antibiotics* **2023**, *12*, 899, doi:10.3390/antibiotics12050899.
89. Sionov, R.V.; Steinberg, D. Targeting the Holy Triangle of Quorum Sensing, Biofilm Formation, and Antibiotic Resistance in Pathogenic Bacteria. *Microorganisms* **2022**, *10*, 1239, doi:10.3390/microorganisms10061239.
